# Pesticides do not significantly reduce arthropod pest densities in the presence of natural enemies

**DOI:** 10.1111/ele.13819

**Published:** 2021-06-23

**Authors:** Arne Janssen, Paul C. J. van Rijn

**Affiliations:** ^1^ Department of Evolutionary and Population Biology Institute for Biodiversity and Ecosystem Dynamics University of Amsterdam Amsterdam The Netherlands; ^2^ Department of Entomology Federal University of Viçosa Minas Gerais Brazil

**Keywords:** biological control, field experiments, insecticides, meta‐analysis, parasitoids, pest control, pest resurgence, population dynamics, predators, transient dynamics

## Abstract

Chemical pesticides remain the main agents for control of arthropod crop pests despite increased concern for their side effects. Although chemical pesticide applications generally result in short‐term decreases of pest densities, densities can subsequently resurge to higher levels than before. Thus, pesticide effects on pest densities beyond a single pest generation may vary, but they have not been reviewed in a systematic manner. Using mathematical predator–prey models, we show that pest resurgence is expected when effective natural enemies are present, even when they are less sensitive to pesticides than the pest. Model simulations over multiple pest generations predict that pest resurgence due to pesticide applications will increase *average* pest densities throughout a growing season when effective natural enemies are present. We tested this prediction with a meta‐analysis of published data of field experiments that compared effects of chemical control of arthropod plant pests in the presence and absence of natural enemies. This largely confirmed our prediction: overall, pesticide applications did not reduce pest densities significantly when natural enemies were present, which concerned the vast majority of cases. We also show that long‐term pesticide effectiveness is underreported and suggest that pest control by natural enemies deserves more attention.

## Graphical abstract

The effects of chemical pesticides on pest densities beyond a single pest generation may vary due to pest resurgence. Using simulations of mathematical predator–prey models, we show that when effective natural enemies are present, chemical pest control will not decrease pest levels throughout a growing season. This prediction was confirmed with a meta‐analysis of published data of field experiments comparing effects of chemical control of arthropod plant pests in the presence and absence of natural enemies.

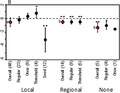

## INTRODUCTION

In addition to their immediate impact on pest species, the side effects of insecticides and acaricides (henceforth pesticides) on humans, beneficial organisms and wildlife have received ample attention in the scientific literature (Bryden et al., 2013; Crall et al., 2018; Desneux et al., 2007; Halstead et al., 2014; Huffaker, 1974; Köhler & Triebskorn, 2013; Rumschlag et al., 2019; Siviter et al., 2018; Yamamuro et al., 2019). In contrast, the effects of pesticides on target pest densities throughout an entire cropping period in the field, which generally includes multiple pest generations, are much less reported and reviewed; effects of pesticides on target pest densities are typically studied during a single generation. Because pesticides are developed and produced with the aim to suppress pest levels in crops, it seems logical to expect that they suppress pest densities at longer as well as shorter time scales. These longer term effects, however, are not so obvious; pesticide applications are also known to result in pest resurgence after an initial reduction of the pest (Guedes et al., 2016; Hardin et al., 1995). One suggested reason for this resurgence is the effects of pesticides on natural enemies of the pest. Simple population models of predators and prey actually predict that long‐term equilibrium densities of pests will increase with pesticide applications when the predators also suffer from increased mortality (Barclay & van den Driessche, 1977; Waage et al., 1985). In such models, the predicted equilibrium pest densities are directly proportional to predator mortality and do not depend on abiotic pest mortality, as was already demonstrated by Volterra (1926). So whereas effective pesticides will obviously reduce pest densities in the short term, pest resurgence is known to occur and population‐dynamical theory predicts that pesticides will not reduce pest levels in the presence of effective natural enemies in the long term.

Except for crops that have a short period of vulnerability to pests, the relevant time scale to assess the effectivity of pesticide use in agriculture is not the short‐term pest dynamics, but the entire cropping or growing season, which often comprises multiple pest generations (Barclay & van den Driessche, 1977; Levins & Wilson, 1980). The dynamics of pests and natural enemies throughout a cropping season will often not be an adequately represented by equilibrium densities (Abrams et al., 1998). Also, agricultural systems are generally complex; they consist of more than one pest and natural enemy species, and natural enemies may feed on other food sources besides the pest. To provide more realistic predictions of the effects of pesticide applications on average pest densities, we simulated the so‐called transient dynamics of pest–natural enemy models. We used models of increasing complexity by adding more species, alternative food and stage structure over multiple pest generations. This theoretical exercise shows that average densities of pests affected by their natural enemies often increase rather than decrease with pesticide applications. To test these predictions, we searched scientific literature for data on pest dynamics with and without pesticide applications and assessed the effect size of pesticide applications over multiple pest generations with meta‐analyses.

## MODELS AND SIMULATIONS

Our simplest (basic predator–prey) model is based on the well‐known Rosenzweig–McArthur (1963) predator–prey model with added pesticide‐induced mortality for both the pest and the natural enemies. The second model represents a tritrophic/food web system, based on the food web model by McCann et al. (1998). The model for this system consisted of a basic trophic level (i.e. the plants), one or two plant pests and a natural enemy. We also used a parameter‐rich stage‐structured model of a well‐studied biological control system (van Rijn et al., 2002), which was validated with greenhouse experiments and of which results are presented in the supporting information. The models did not include spatial structure, assuming that agricultural fields harbour well‐mixed populations of pests and natural enemies.

### Basic predator–prey model

The differential equations of pest (*N*) and natural enemy (*P*) densities of our version of the Rosenzweig–McArthur (1963) model are as follows:

$$\frac{\mathrm{dN}}{\mathrm{dt}}=rN\left(1‐\frac{N}{K}\right)‐\frac{\mathrm{aNP}}{N+D}‐pN,$$

$$\frac{\mathrm{dP}}{\mathrm{dt}}=\frac{\mathrm{acNP}}{N+D}‐mP‐pqP,$$

where *r* is the pest growth rate (0.166/day), *K* is the carrying capacity (1000 pest individuals /m^2^), *a* is the maximum predation rate (4 prey/predator/day), *D* is the pest half‐saturation density of the predator Type II functional response (1500 prey/m^2^), *p* is the pesticide‐induced mortality (variable, 1/day), *c* is the rate of conversion of consumed pests into predator reproduction (0.375 predators/prey), *m* is the natural predator mortality (0.1/day). Model parameters were based on studies of a pest–predator system by van Rijn et al. (1995, 2002) for thrips and predatory mites at 22°C. The pesticide‐induced mortality (*p*) is given as a constant rate here, but was simulated as four different application methods: (1) a pulse application during 1 day once every 14 days; (2) a pulse application during 1 day at intervals varying from daily to once per season (100 days), (3) a pulse application during 1 day, but only applied above a threshold density of the pest (hence, intervals depending on pest density) and (4) applications as a constant mortality factor, representing pesticides with a longer, systemic effect or the treatment of seeds with pesticides. For simulations of threshold pesticide applications, we assumed a monitoring interval of 6 days, after which pest densities were assessed and pesticide‐induced mortality was simulated during 1 day as above, but only when pest densities surpassed a threshold density (200 pest individuals/m^2^). Other monitoring intervals gave qualitatively similar results.

The parameter *q* (variable, ratio) scales the pesticide‐induced mortality of the natural enemies relative to that of the pest, with *q* = 0 for completely selective pesticides that impose mortality only on the pest, 0 < *q* < 1 for somewhat selective pesticides and *q* > 1 for pesticides that induce higher mortality on the natural enemies than on the pest. Simulations started with low pest and natural enemy populations, as is usually the case at the beginning of a cropping season, and lasted for five pest generations, which is within the range reported in the field studies reviewed below. Simulations lasting half that period (2.5 pest generations) gave qualitatively similar results. The values of pesticide‐induced pest mortality (*p*) used in the analysis were based on the range of instantaneous pesticide‐induced mortalities in the field found in 10 studies (15 pest species, 18 active ingredients or combinations) on instantaneous mortality data (i.e. 0–2.7/day, average 0.8/day, Table S1).

The generation time of the pest was taken as 20 days (van Rijn et al., 1995); hence, simulations lasted for 100 days, coinciding with the average numbers of pest generations in the publications reviewed below (average 4.9 generations and 102.4 days). Initial densities of the standard simulations were 50 pest and 10 natural enemy individuals per m^2^, but were also varied. We also analysed a version of this model with parameter values for aphids and their natural enemies (Trumper & Holt, 1998), which showed similar results (not shown).

### Tritrophic/food web model

Most agricultural food webs consist of more than one predator and pest, and we, therefore, also used a more complex food web, based on that by McCann et al. (1998). The version of the model used here consists the basic trophic level (i.e. plants), one or two herbivores that feed on this plant (with one being the pest) and one predator that feeds on both herbivores. The two herbivores, thus, interact through competition for the plant and through a shared predator (so‐called apparent competition or apparent mutualism, Abrams et al., 1998; Holt, 1977).

Our version of the model is as follows:

$$\begin{array}{cc} \frac{\mathrm{dR}}{\mathrm{dt}} & =R\left(1‐\frac{R}{K}\right)‐\frac{x_{N1}y_{N1}RN_{1}}{R+R_{01}}‐\frac{x_{N2}y_{N2}RN_{2}}{R+R_{02}},\\ \frac{dN_{1}}{\mathrm{dt}} & =‐x_{N1}N_{1}\left(1‐\frac{y_{N1}R}{R+R_{01}}\right)‐\frac{\Omega x_{P}y_{P}N_{1}P}{\Omega N_{1}+\left(1‐\Omega \right)N_{2}+N_{0}}‐pN_{1},\\ \frac{dN_{2}}{\mathrm{dt}} & =‐x_{N2}N_{2}\left(1‐\frac{y_{N2}R}{R+R_{02}}\right)‐\frac{\left(1‐\Omega \right)x_{P}y_{P}N_{2}P}{\Omega N_{1}+\left(1‐\Omega \right)N_{2}+N_{0}}‐psN_{2},\\ \frac{\mathrm{dP}}{\mathrm{dt}} & =‐x_{P}P\left(1‐\frac{\Omega y_{P}N_{1}+\left(1‐\Omega \right)y_{P}N_{2}}{\Omega N_{1}+\left(1‐\Omega \right)N_{2}+N_{0}}\right)‐pqP, \end{array}$$

where *R* is the plant density, *N*_1_ is the pest density, *N*_2_ is the alternative prey (herbivore) density and *P* is the predator density, *K* is the carrying capacity of the resource, *R*_01_, *R*_02_ and *N*_0_ are half‐saturation densities of the resource and the herbivores, *x_i_* and *y_i_* are the metabolic rate and the ingestion rate of species *i*, Ω is the preference of the predator for prey *N*_1_ relative to *N*_2_, *p* is the pesticide‐induced mortality of the pest (*N*_1_) and *q* and *s* are the pesticide‐induced mortality of the predator and alternative prey, respectively, both relative to the mortality of the pest. The parameters are a bio‐energetic interpretation for invertebrates of a version of the Rosenzweig–MacArthur model (McCann et al., 1998; McCann & Yodzis, 1994; Yodzis & Innes, 1992). Parameter values were taken from McCann et al. (1998); some were adapted to ensure coexistence of the predators (*P*) with either of the two prey species separately (*K* = 1, *x_N_*_1_ = 0.201, *x_N_*_2_ = 0.2, *x_P_* = 0.08, *y_N_*_1_ = 2.009, *y_N_*_2_ = 2.01; *y_P_* = 5, *R*_01_ = 0.1625, *R*_02_ = 0.16129 and *N*_0_ = 0.5). For these parameter values and without pesticide applications (*p* = 0), the system shows more or less complex fluctuations in which all four species coexist for at least 5000 time steps. We assumed no preference of the predator for either of the two prey species (Ω = 0.5). See Yodzis and Innes (1992) and McCann et al. (1998) for further details and units. Pesticide applications were simulated as above and initial densities were *R* = 0.1, *N*_1_ = 0.2, *N*_2_ = 0 (no alternative prey) or 0.2 (with alternative prey) and *P* = 0.1. Threshold densities were 0.5 individuals. Because the long‐term dynamics of this system depends to a large extent on initial densities, we also investigated their effects. Simulations again lasted for 100 days. All simulations were run in R (R Core Team, 2019) with the package deSolve (Soetaert et al., 2010, 2012).

### Modelling results

As an illustration of the long‐term effects of pesticides on pest densities, it serves to study the positive equilibrium density of the pest in the Rosenzweig–McArthur predator–prey model. It is obtained by calculating the natural‐enemy isoclines, which gives as positive pest equilibrium in the presence of natural enemies N* = (*m* + *pq*)*D*/(*ac* – *m* – *pq*). This pest equilibrium is solely determined by predator characteristics (Volterra, 1926), and it increases with increasing *p* or *q*, hence applying pesticides in the presence of natural enemies that are sensitive to them will result in higher equilibrium pest densities, in agreement with earlier results (Barclay, 1982; Waage et al., 1985). Notice that if *q* = 0, the original equilibrium density of the pest without pesticides is obtained. This means that equilibrium pest densities with a completely selective pesticide that does not kill natural enemies are not reduced or increased by pesticide use. Hence, natural enemies, and not the pesticides, determine the equilibrium densities of the pest (Barclay, 1982; Barclay & van den Driessche, 1977; Supporting information S1).

It is well‐known that the original Rosenzweig–McArthur model can give rise to cycles of increasing amplitude with increasing carrying capacity *K* (the so‐called paradox of enrichment (Rosenzweig, 1971)). Adding constant pesticide‐induced mortality can then stabilise these unstable dynamics, but at higher average pest levels than with unstable dynamics (Supporting information S1, Figure S1).

**FIGURE 1:**
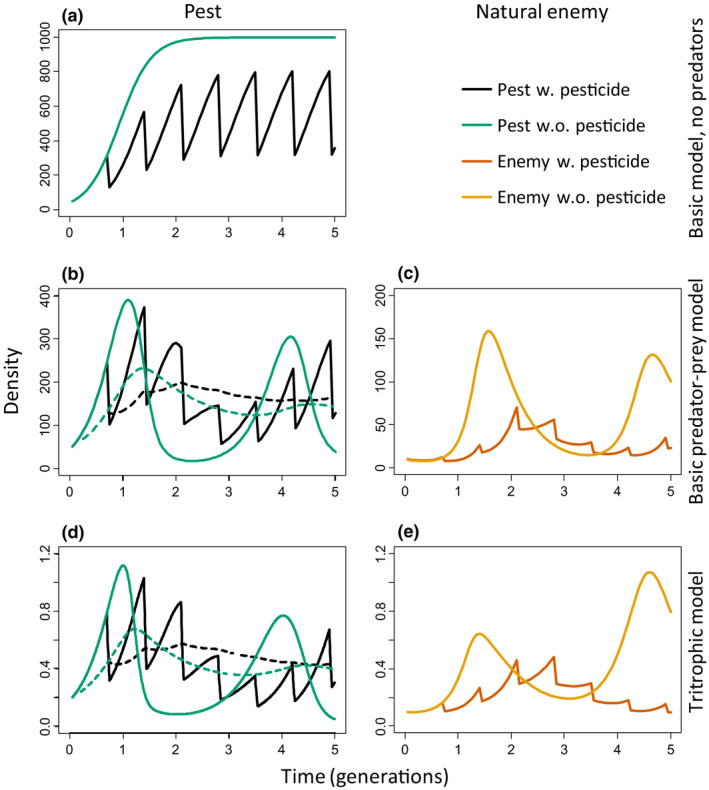
Representative medium‐term dynamics of pest and natural enemy densities (five pest generations). Shown are densities (vertical axis) as function of time (horizontal axis, in pest generations). (a) Pest densities of a basic Rosenzweig–McArthur model without predators and without pesticides (green curve) or with pesticides applied every 14 days (*c*. 0.7 pest generation, black curve). (b) Pest densities of the same model, but with predators, green and black curves as in (a). Pest resurgence can be seen by the spiked black curve being higher than the smooth green curve between 1.5–3.5 and after 4.5 generations. This results in higher average pest densities as indicated by the cumulative average densities over zero to five generations (dashed curves). (c) Predator densities of the same model, orange and brown curves are predator densities without and with pesticide application respectively. (d) Pest densities of a tritrophic model consisting of a plant, a pest and a natural enemy, curves as in (b), plant densities not shown. Pest resurgence occurs between c. 1.2–3 and after 4.5 generations. (e) Predator densities of the tritrophic model. Pesticide‐induced pest mortality was *p* = 1/day and natural enemy mortality half of that (*q* = 0.5). See text for other parameter values

To estimate the effects of pesticides during a crop growing season, we ran simulations of dynamics over five pest generations. These showed that pulsed pesticide applications in the absence of natural enemies resulted in the expected decrease in pest densities (Figure 1a, reductions of the pest through periodic pesticide application results in the spiked black curve). With natural enemies, pesticide applications initially decreased pest densities even further (Figure 1b, black curve), but later resulted in higher pest densities (i.e. pest resurgence) than those obtained without pesticides (Figure 1b, green curve), even when the natural enemies were half as sensitive to the pesticide as the pest (Figure 1c). A similar phenomenon of increased pest densities after pesticide applications was observed with the tritrophic model (Figure 1d,e). Hence, there is an initial decrease in pest densities due to pesticide applications (Figure 1b,d), followed by an increase relative to the densities without pesticide applications, and what matters now is how this affects average pest densities over time. The interrupted black and green curves in Figure 1b,d give cumulative average pest densities with and without pesticides, showing that average pest densities with pesticides were lower than without pesticides up to 1.65–1.95 pest generations, but were consistently higher when averaged over longer periods. Overall, the simulations indicate that pest resurgence is likely a common phenomenon when effective natural enemies of the pest are present.

The question now is whether this resurgence leads to higher average densities of the pest during a growing season, as this will result in more damage in most systems. Because equilibrium densities may not be an adequate representation of average densities in unstable nonlinear systems (Abrams et al., 1998, see also Supporting Information S1, Figure S2), we ran simulations as shown in Figure 1 and averaged the densities over five pest generations. As can be seen in Figure 1b,d, the difference in average pest densities with and without pesticide applications after five generations did not differ as much as for shorter simulations (interrupted lines), so the effects of pest resurgence averaged over five pest generations are less pronounced than for shorter periods (e.g. three generations).

**FIGURE 2:**
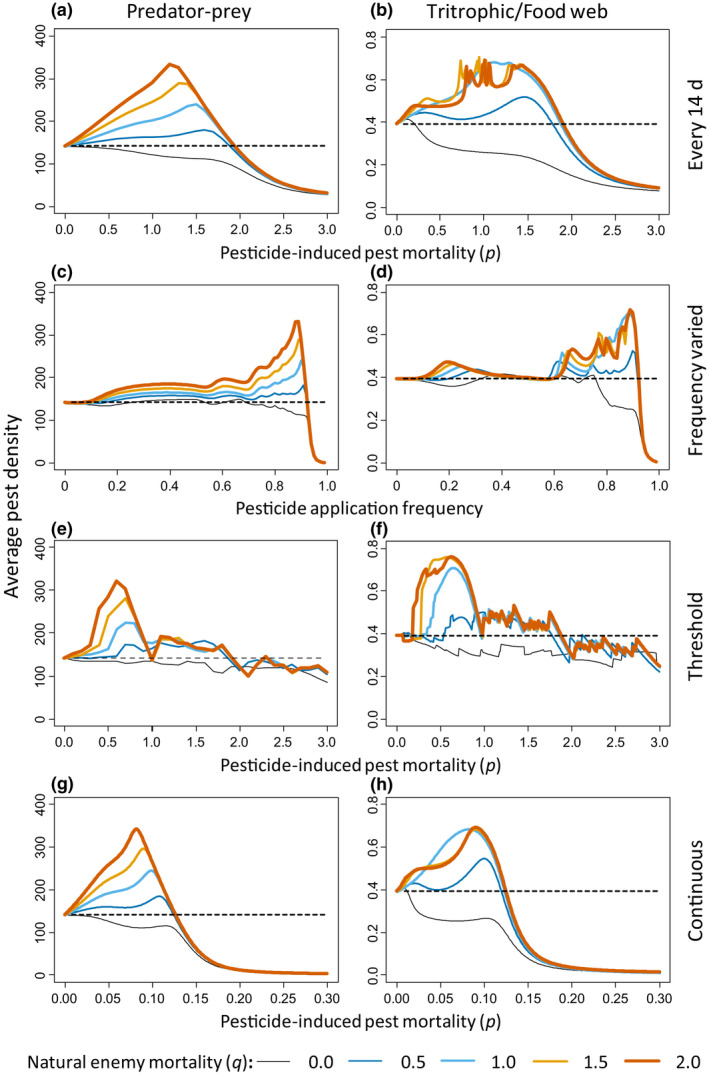
Average pest densities (left vertical axis of each panel) over five pest generations (i.e. 100 days) of a predator–prey model (left column) and a food web model without alternative prey (right column) as a function of pesticide‐induced pest mortality (*p*) or pesticide application interval (horizontal axes). Each row represents a different pesticide application method, indicated to the right of the rows. Top row (a, b): Pesticide application is every 14 days; the mortality of the pest due to pesticide application varies from 0 to 3 (horizontal axes). Second row (c, d): The pesticide‐induced pest mortality rate is constant (*p* = 1/day), but the application frequency (horizontal axes) varies from none (0) to daily (1): a frequency of 0.86 means an application every 14 days. Third row (e, f): Pesticide application is according to a threshold pest density, the mortality of the pest due to pesticide application varies from 0 to 3 (horizontal axes). Bottom row (g, h): pesticide is applied continuously, pest mortality (horizontal axes) varies from 0 to 0.3 (horizontal axes). Per panel, curves with different colours and thickness refer to different pesticide‐induced natural enemy mortality relative to pest mortality: thin, black: *q* = 0; blue: *q* = 0.5; light blue: *q* = 1; orange: *q* = 1.5; thick, brown: *q* = 2. Increasing line thickness corresponds to increasing enemy mortality. The pest density obtained without pesticide application is given as reference by black dashed horizontal lines

**FIGURE 3:**
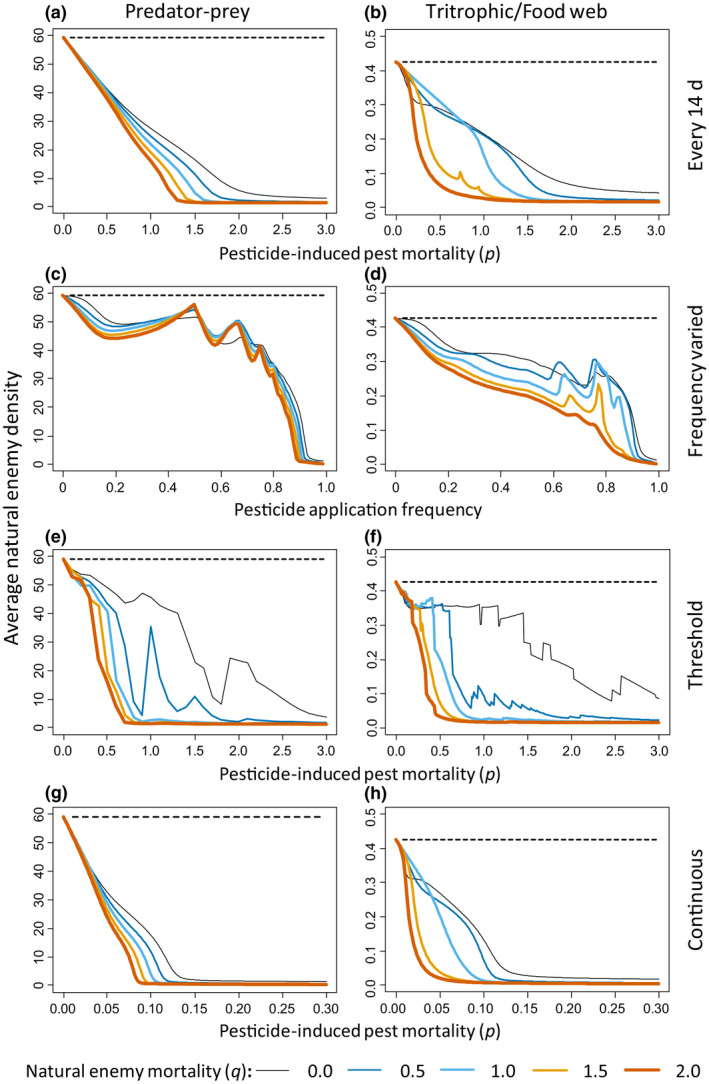
Average predator densities corresponding to the average pest densities are presented in Figure 2. Horizontal dashed lines give predator densities without pesticide application for reference. See legend to Figure 2 for further explanation

We first simulated variations in pesticide‐induced pest mortality (0 < *p* < 3) and natural enemy mortality (0 ≤ *q* ≤ 2) with pulsed pesticide applications at intervals of 14 days. For low pesticide‐induced pest mortality rates (0 ≤ *p* ≤ 1.9), the basic predator–prey model and the tritrophic model showed higher pest densities, averaged over the growing season, with pesticide applications (Figure 2a,b, coloured curves) than without pesticides (Figure 2a,b, dashed black lines), except when there was no pesticide‐induced mortality of the natural enemies (*q* = 0, Figure 2a,b, black curves). At higher pest mortality rates (*p* > 1.9), predator populations went extinct (Figure 3a,b), and pesticide applications resulted in reductions of average pest densities to below those obtained without pesticide (Figure 2a,b, curves are below dashed black line for *p* > 2.0), in agreement with simulations without natural enemies (Figure 1a). Not surprising, higher predator mortality (*q*) relative to the pest mortality resulted in higher mean pest densities (Figure 2a,b).

Second, varying the pesticide application frequency (with an intermediate pesticide‐induced mortality *p* = 1 day) also resulted in higher average pest densities with pesticides than without pesticides when natural enemies suffered from pesticide‐induced mortality (*q* > 0) for all but the highest frequencies (Figure 2c,d), the latter coinciding with strong reduction or extinction of the predator population (Figure 3c,d). The same general patterns of increased average pest densities as with pulsed pesticide applications (Figures 2a,b and 3a,b) were found with threshold‐based (Figures 2e,f and 3e,f) and continuous (or systemic) pesticide applications (Figures 2g,h and 3g,h). One difference is that with threshold and continuous applications, peaks in average pest densities occur at lower values of pesticide‐induced pest mortality than with pulsed applications, which coincides with a decrease in average natural enemy densities (Figure 3).

Transient, short‐term dynamics are affected by the initial densities of pests and natural enemies. We, therefore, simulated dynamics of the models starting at different initial pest–enemy ratios and various pesticide‐induced mortality rates, assuming constant pesticide application (Figure S3). For the basic predator–prey model, there were only small effects of initial densities, without affecting the effects of pest mortality (*p*) on pest densities (Figure S3a,c,e). For the food web model (Figure S3b,d,f), effects of initial pest–enemy ratios were more pronounced when natural enemies also suffered mortality from pesticides (*q* > 0). However, for all initial densities in both models, pesticide‐induced natural enemy mortality (*q* > 0) resulted in increased pest densities for low to intermediate pesticide‐induced mortality of the pest. Higher pesticide‐induced mortality again caused the (near) extinction of the natural enemies (data not shown), resulting in decreases in pest densities with increasing pest mortality, irrespective of the initial densities.

Analysis of the stage‐structured pest–natural enemy model (Supplementary information S2) shows somewhat more complex results: resurgence occurred only for the pest stage that was vulnerable to predation, and densities of invulnerable stages decreased with increasing pesticide‐induced pest mortality (Figure S4a). As a result, the average pest densities including all stages did not increase for low to intermediate mortality of the natural enemies (Figure S5). Because the vulnerable stage in this model was very short, we investigated the effect of longer periods of prey vulnerability (Supplementary information S2, Figure S4b), in which case pesticide application often did not result in decreased average pest densities (Figure S6), and resulted in increased pest densities when natural enemies suffered high mortality from the pesticide (Figure S6). Thus, the duration of the period that the pest is vulnerable to the natural enemies influences the effect of pesticides on pest densities.

In conclusion, the modelling exercise shows that pesticide applications in the presence of effective natural enemies likely lead to increased average pest densities when the enemies suffer low to intermediate pesticide‐induced mortality. Pesticide applications only lead to decreased pest densities when natural enemies are near extinction. Applying pesticides with low to intermediate pest mortality rates or application rates even results in increased average pest densities. We subsequently tested this prediction by reviewing the literature on effects of pesticides during several pest generations in the field in the presence and absence of populations of effective natural enemies.

## META‐ANALYSIS

### Literature selection and analysis

The Web of Science was searched for publications in English from 1975 to March 2020 using the search terms: “(pest control) AND (field) AND (pesticide OR insecticide OR acaricide)” (3890 results); “(pest) AND (chemical control) AND (field) NOT (pesticide OR insecticide OR acaricide)” (813 results); “(pest) AND (transgenic) AND (field) AND (pesticide OR insecticide OR acaricide)” (371 results) and “(pest resurgence) AND (field)” (68 results). We also included studies mentioned in Marvier et al. (2007) and Wolfenbarger et al. (2008) and studies referred to in the papers found above. Only studies including above‐ground arthropod plant pests were considered.

The studies had to have at least one treatment without and one with synthetic pesticides and had to present the averages, sample sizes and standard errors or standard deviations of the pest densities on the crop plants. Studies with trap counts were excluded because the efficiency of these traps was difficult to assess. Because of the cumulative nature of damage, the few studies presenting damage data and no pest densities were excluded. Treatments had to have been applied in replicates and in blocks in the open field in the same general area (i.e. no cages or greenhouses). Only natural infestations of the pests were considered, and only studies in which no natural enemies were released. Thus, chemical pest control was compared with natural control. Treatments involving transgenic plants or “natural” pesticides were excluded. Most of the studies found were omitted because their titles and abstracts made clear that they did not meet our criteria, resulting in 444 publications that were further analysed.

Our model simulations show that the effect of pesticides on pest populations depends on the moment of evaluation. For example, Figures 1b,d show that sampling after one pest generation results in negative effects of pesticides on pest densities (black curve) relative to the control without pesticides (green curve), but sampling after two generations shows positive effects. It is, therefore, important that pest populations were sampled repeatedly during several pest generations. Studies, therefore, had to include at least two generations of the pest and had to present either repeated measures or long‐term averages of repeated measures of pest densities. We searched the internet for monthly average temperature data of the research sites (https://www.meteoblue.com) and calculated the average temperature during the experiments as accurately as possible. We also searched the internet for data on generation times of the pest species, preferably at several different temperatures and from several different peer‐reviewed sources, and used this, combined with local temperature data to estimate the generation time of the pests in the field. The durations of the experiments were scaled to these pest generation times. This resulted in further loss of data because several experiments proved shorter than two pest generations. Consequently, there were 25 publications from which we could extract data that were suitable for the meta‐analysis (Table S2). These publications either presented repeated measurements of pest densities (a total of 67 time series) or average pest densities over at least two pest generations (96 data sets), including in total 33 pest species and their various natural enemies (Table S2). Mean densities, sample sizes and standard deviations were used to calculate effect sizes, expressed as the standardised mean difference (i.e. Hedges *g*; Hedges, 1981) and unbiased sampling variances for all data sets.

We assessed whether natural enemies were present or not, either from the same study (scored as “local”), from a different study in the same country, province or state (“regional”), or as absent. It was impossible to classify the natural enemies according to their effectiveness because the publications often did not provide sufficient information on this topic. The method of pesticide application was scored as “once” when pesticides were applied only once, “regular” when pesticides were applied more often, “threshold” when they were applied according to some threshold pest density or damage rule and “seed” when seeds were coated with systemic pesticides before being planted.

Some publications presented data in tables, but most data were presented in figures. In the latter case, we first approached the authors with a request for the original data, and in case of no response we extracted the data from published figures with sufficiently high resolution with WebPlotDigitizer (https://apps.automeris.io/wpd/) (Rohatgi, 2017). Plots were made with extracted data and were superposed on the published plots to ensure the accuracy of this process.

The data were analysed with multivariate mixed‐effects models (the rma.mv function of Metafor; Viechtbauer, 2010) with presence of natural enemies, duration of the experiment (in number of pest generations) and pesticide application method as moderators (factors) (Viechtbauer, 2010). Reductions of pest densities due to pesticide use relative to the untreated control are presented as negative effect sizes. For analysis of the time series, time within individual time series was used as random factor and a continuous‐time autoregression structure was used (Viechtbauer, 2020). For average data, the individual study was used as random factor. Many studies had clusters of one treatment without pesticides as control and various treatments with different pesticides; hence, we needed to use the control in several comparisons, but corrected for this by calculating robust tests and confidence intervals with the robust function of Metafor (Viechtbauer, 2010). We determined the significance of moderators and interactions with deletion tests. Contrasts among moderator levels were assessed by aggregating levels followed by likelihood ratio tests comparing different models. All analyses were carried out with the package Metafor in R (“devel” version 2.1‐0, Viechtbauer, 2010).

### Results of the meta‐analysis

**FIGURE 4:**
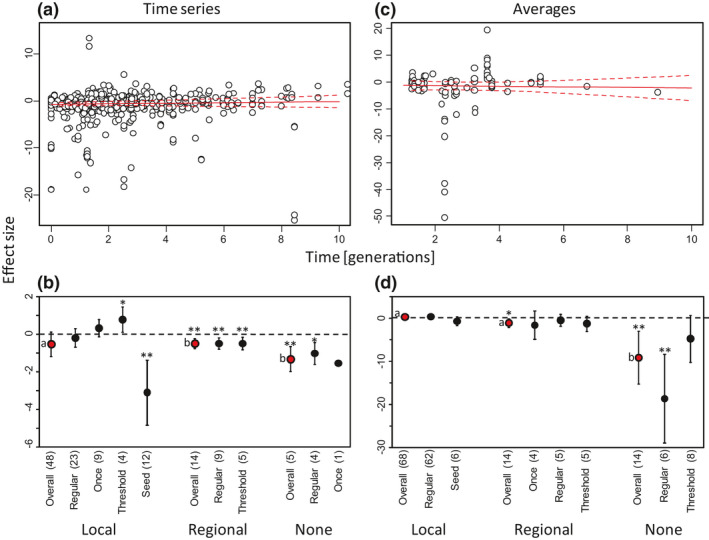
The effect of pesticides on pest densities in field experiments with time series of pest densities (left column) and average pest densities per season (right column). In (a), each point shows the effect size (Hedges g) of one time point of time series of pest densities in time; in (c), each point represents a seasonal average as function of half the length of the total season. Positive values mean a positive effect of pesticides on pest densities. Overall, the trend through time (drawn red line) is close to no effect (zero). (b) and (d) show the effect of the presence of natural enemies and the pesticide application method on pesticide efficacy. (b) shows effects on repeated measures of studies reporting time series; (d) shows effects of studies presenting long‐term average pest densities. Local: natural enemies were present locally; Regional: natural enemies were reported from the same region but not in the publication evaluated; None: no record of natural enemies. Within each of these enemy presence categories, the overall effect (red dots) and the effect per pesticide application method (black dots) are given. Once: pesticides were applied once during the experiment; Regular: pesticides applied several times; Threshold: pesticides were applied according to some damage or pest density threshold; Seed: pesticides were applied to the seeds before planting. Dashed lines in (a) and (c) and error bars in (b) and (d) are 95% confidence intervals. Numbers between brackets after the horizontal axis labels in (b) and (d) give the number of cases, letters next to data points indicate significant differences between overall effects, asterisks indicate significance of the effect: *p < 0.05; **p < 0.01

The meta‐analysis of time series of pest densities showed that the effect size of pesticide applications was not significantly affected by the duration of the study (Figure 4a: Likelihood Ratio Test (LRT) = 1.61, d.f. = 1, *p* = 0.20). Especially during the first generations of the pest, the effect of pesticide application on pest densities varied considerably, from strongly positive to strongly negative (Figure 4a), in agreement with the dynamics illustrated in Figure 1b,d. Overall, the average effect size through time was very close to zero (i.e. no effect of pesticides compared to the control, Figure 4a). The effect size differed significantly with the presence of natural enemies (LRT = 11.0, d.f. = 2, *p* = 0.004). The overall effect of pesticides on pest densities was small and did not differ significantly from zero when natural enemies were observed locally (Figure 4b), which concerned the majority of the time series (71.6%). In the presence of natural enemies, threshold pesticide applications resulted in a significant positive effect on pest densities, whereas systemic seed treatments resulted in a significant and strong negative effect. In agreement with the predictions, pesticides had a significant negative effect on pest densities when no natural enemies were reported locally or regionally (Figure 4b), which concerned 7.5% of all cases analysed. When natural enemies were reported from the region of the experimental site but not locally (i.e. not in the publication evaluated), pesticides had a small but significantly negative effect on pest densities (Figure 4b).

The effect of duration of the study was also not significant for studies presenting long‐term averages of pest densities (Figure 4c, LRT = 0.99, d.f. = 1, *p* = 0.32), which is in agreement with the time‐series data (Figure 4a). Effects varied strongly among cases (Figure 4c), and the average effect size through time was again not significantly different from zero (i.e. no effect of pesticides). The effect of pesticide applications on pest densities again varied significantly with the presence of natural enemies (LRT = 19.0, d.f. = 1, *p* < 0.0001). In the majority of cases (70.8%), natural enemies were observed locally, and the overall effect of pesticide application on pest densities was not significantly different from zero (Figure 4d) for all pesticide application methods investigated. The overall effect of pesticides on pest densities was again negative when no natural enemies were reported (Figure 4d), especially with regular pesticide applications, but not with threshold applications. When natural enemies were reported present in the region but not in the particular study, the overall effect of pesticides on pest densities was slightly, but significantly negative, although none of the pesticide application methods resulted in significant decreases in pest densities (Figure 4d). See Supporting Information S3 and Table S3 for further analysis of the literature.

## DISCUSSION

We show that pesticides have significant negative effects on pest densities when effective natural enemies of the pests are absent, but not when they are present. This is because these effective enemies reduce pest populations and the added effect of pesticide‐induced pest mortality can, therefore, only be small. Without effective natural enemies, pest densities are higher and pesticides can have a larger effect on them. When the densities of natural enemies are reduced by the pesticide, pest densities escape control by the natural enemies and can resurge, resulting in higher average pest densities.

**FIGURE 5:**
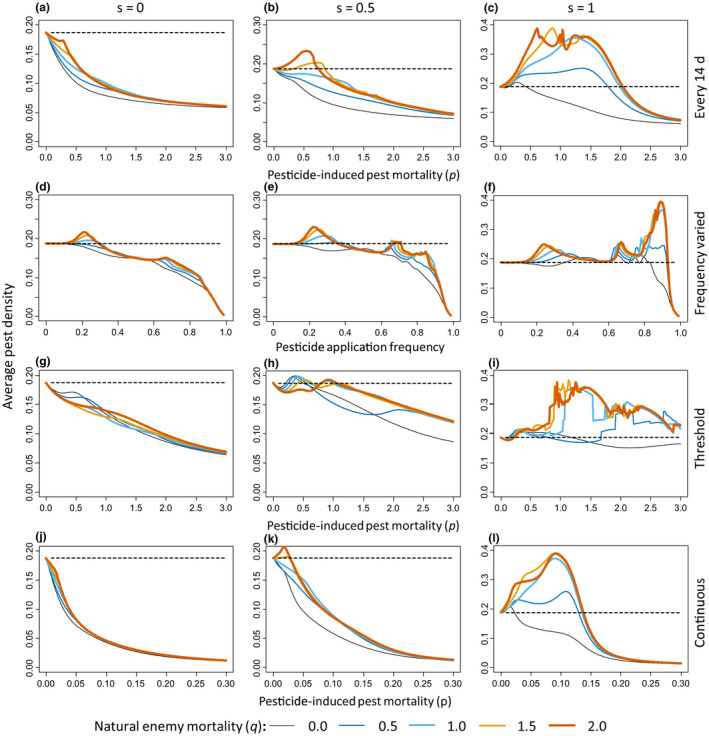
Average pest densities of simulations of the tritrophic/food web population model (five generations) with an alternative prey. Shown are average pest densities with no pesticide‐induced mortality of the alternative prey (*s* = 0, left‐hand column), mortality of the alternative prey being half that of the pest (*s* = 0.5, middle column) or the same as that of the pest (*s* = 1, right‐hand column). Top row (a–c): Pesticides applied every 14 days. Second row (d–f): Interval of pesticide application varied, with pesticide‐induced pest mortality (*p*) = 1/day. Third row (g–i): Pesticides applied at a threshold pest density of 0.5. Bottom row (j–l): Pesticides applied continuously. See legend to Figure 2 for further explanation

Several factors have repeatedly been mentioned as causes for pest resurgence: (1) the pesticide being more toxic to the natural enemies than to the pest; (2) extinction of the local natural enemy population as a result of pesticide use; (3) a stimulatory effect of sub‐lethal doses of pesticides (hormesis, Dutcher, 2007; Guedes et al., 2016; Hardin et al., 1995; Morse, 1998). When mortality of natural enemies induced by pesticide applications was taken as half that of the pest, our simulations show that natural enemies did not go extinct, and no hormesis was included, yet resurgence occurred (Figure 1). We, therefore, conclude that the most parsimonious explanation for pest resurgence is increased mortality of effective natural enemies, and this mortality does not need to be higher than that of the pest. We suggest that the mechanism behind pest resurgence is that the pest suffers from the pesticide at intermediate mortality levels, but is simultaneously partially released from predation because the natural enemy population suffers both from pesticide‐induced mortality and from limited food availability due to reduced pest densities. The importance of this limited food availability is shown with simulations with alternative prey or alternative food. Simulations with the tritrophic/food web model show that the effects of pesticides on the natural enemies are reduced in the presence of alternative prey if the latter is not affected by the pesticide (Figure 5, *s* = 0). The natural enemies still suffer from pesticide‐induced mortality (Figure S7), but the presence of alternative prey results in higher densities of the natural enemies (Figure S7a,d,g,j, cf Figure 3b,d,f,h), leading to lower pest densities (Figure 5a,d,g,j, cf. Figure 2b,d,f,h). With increasing pesticide‐induced mortality of the alternative prey (Figure 5, *s* = 0.5), predators have fewer alternative prey, densities of the natural enemies are reduced (Figure S7) and pest densities are controlled less (Figure 5b,e,h,k), sometimes reaching higher densities than without pesticides. When the mortality of the alternative prey equals that of the pest, natural enemy densities are similar to those without alternative prey (cf. Figure S7c,f,i,l with Figure 3b,d,f,h), and pest densities are practically the same as without alternative prey (*s* = 1, Figure 5c,f,i,l, cf. Figure 2b,d,f,h). Adding pollen as alternative food for the natural enemies in the stage‐structured model also resulted in somewhat reduced pest densities as a result of increased pesticide‐induced mortality, but not when pesticides caused high mortality of the natural enemies (Supporting information S4, Figure S8).

The consequences of alternative prey/food for biological pest control are relatively well studied, and, depending on the type of dynamics (fluctuating or stable) and on the time scale (short‐term vs. long), can range from positive effects of increased densities of one herbivore species on densities of the other species (apparent mutualism, Abrams et al., 1998; van Maanen et al., 2012) to negative effects on herbivores (apparent competition, Bompard et al., 2013; Emery & Mills, 2020; Holt, 1977; Holt & Bonsall, 2017; Karban et al., 1994; Langer & Hance, 2004; Liu et al., 2006; Messelink et al., 2008; Muñoz‐Cárdenas et al., 2017; van Rijn et al., 2002). In the simulations presented here, the effects of alternative food and prey on the pest were mostly negative, hence, can be classified as apparent competition.

In general, the model simulations showed higher average pest densities after pesticide applications when natural enemies were present, but we did not detect such overall positive effect in the meta‐analysis. Of the time series and seasonal averages with natural enemies present, 46.6% showed a positive effect, indicating that the presence of natural enemies did not always result in a positive effect of pesticides on pest densities. There are several explanations for this difference between model predictions and experimental results. First, the experimental and control plots were often situated at close distance from each other, and after pesticides application, the treated plots can quickly be recolonised by pests, but also by natural enemies from the control plots and resurgence is then less likely to occur. Second, the models assumed that natural enemies were effective in reducing pest densities, but the potential effectiveness of the natural enemies in the experimental studies was often unknown. If natural enemies were present, but not effective, resurgence would not occur, and pesticide applications would result in lower average pest densities relative to the control. Third, the results of the meta‐analysis can have been affected by uncertainty about the presence of natural enemies. In particular, the absence of a record of natural enemies is no proof of their absence. In all five cases classified as “None” in the time series (Figure 4b), the authors explicitly mentioned their absence. However, in 11 of the 14 cases of “None” with long‐term averages (Figure 4d), there was no such evidence. We, therefore, repeated the meta‐analysis of these data, including them in the group where natural enemies were present regionally. This resulted in minor changes in the effect sizes of the cases with natural enemies present regionally (results not shown). Although the effects of pesticides in cases without natural enemies were all negative, the overall effect size was no longer significantly different from zero because of the low number of cases (i.e. 3). Fourth, our models concerned single species of natural enemies, whereas generally, various species of generalist and specialist enemies were present in the field (Table S2), and some of these generalist species may have been feeding on other prey and pest species that were less affected by the pesticides, which decreases the pest resurgence effects (Figure 5, Supplementary information S4). Related to this is the effect of the relatively small plot sizes, which may have allowed the natural enemies to forage on pests and other alternative food outside the treated plots (see further discussion on the effects of plot sizes below). Lastly, the model simulations predict that average pest densities decrease at pesticide levels that bring the enemies near extinction (Figures 2 and 3). We did not have sufficient information on the condition of the natural enemy populations to verify when this was the case in the field studies.

Although the meta‐analysis did not show indications for higher pest levels due to pesticide applications, it did largely support the prediction that pest densities were not reduced by pesticide applications in the presence of natural enemies (Figure 4). The only exceptions were 12 time series (from three publications) with seed treatments out of a total 48 time series (Figure 4b), but no such trend of decreased pest densities with seed treatments was seen in the six studies presenting average densities (Figure 4d).

### Time scales

We specifically simulated population dynamics spanning several pest generations, but even longer term dynamics may be relevant for perennial agricultural systems such as orchards, although most of these often also exhibit seasonal dynamics and do not reach equilibria (Lester et al., 1998; Pascual‐Ruiz et al., 2014; Prischmann et al., 2005). Nevertheless, equilibrium dynamics may be relevant for some systems. As explained in the introduction and Supplementary Information S1, dynamics of simple predator–prey models show higher equilibrium pest densities with increased pesticide‐induced mortality; hence, long‐term, stable dynamics would show the same trend as the dynamics presented here.

All models presented here show a relatively fast numerical response to increases in pest density, such as in predatory mites and natural enemies of aphids (coccinellids, syrphids; Trumper & Holt, 1998). However, some natural enemies, such as ground beetles and spiders, have one generation per year and may show limited numerical responses at the time scale of a cropping season. If such natural enemies are effective and sensitive to pesticides, it is obvious that it will take their populations longer to recover from pesticide applications than enemies with shorter life cycles, and we expect that pest resurgence will then occur for a longer period. Furthermore, pests are often attacked by various natural enemies, with some of them showing a numerical response within one growing season, and others not, and research on the effects of pesticides on the control of pests by various species of natural enemies, therefore, deserves further attention.

Pesticides will be applied to have a negative effect on pest densities, irrespective of the presence of natural enemies, but neutral effects of pesticides and pest resurgence are theoretically expected to occur at longer time scales when effective natural enemies are present. The effects of pesticides should, therefore, be evaluated in field studies over longer periods. Furthermore, it is important to monitor the effects of pesticides with repeated measurements of pest densities throughout a cropping season, because snapshots of densities at a particular time may show effects that differ from the long‐term trends (Figure 1b,d). In addition, these experiments need to be done on larger spatial scales, comparable to commercial fields, because the dynamics of pests and natural enemies in such fields will be affected by the (differences in) migration of pests and natural enemies. In fact, the low number of published field studies of sufficiently long duration and with repeated measures is cause for concern, especially because pest resurgence may invert the trends observed in short‐term, small‐scale studies.

### Spatial scales

The average size of the fields in the experimental studies was small relative to commercial fields (Table S2), and this may have affected the effects of pesticides in these studies. On the one hand, natural enemies will take more time to fully recolonise larger fields from surrounding habitats (Jepson & Thacker, 1990; Thomas et al., 1990), and pests will have more opportunities to escape from natural pest control (Gagic et al., 2019), resulting in increased pest resurgence. On the other hand, large commercial fields may lack natural enemies at the start of the growing season, and pesticides applied before these enemies arrive will reduce pest densities. Moreover, natural enemies will vary in their dispersal capacities, and specialist natural enemies may be less successful in colonising fields than generalists, because the former are dependent on prior colonisation by the pest, whereas the latter can also feed on other food sources present in the crop field. To further investigate the effect of field size, we analysed whether plot size affected the effect of pesticide applications, which did not show a significant effect of plot size (Supporting information S3). However, the size range of the experimental plants was limited; hence, it is likely that effects of pesticides will be affected by larger plot sizes.

### Stage structure

Simulations of the stage‐structured population model showed that the existence of pest stages that are invulnerable to natural enemies affects the effects of pesticides on total pest densities, with pesticides being more effective with pests with short vulnerable stages (Figures S4–S6). Many pests have stages that are invulnerable to some natural enemies (Murdoch et al., 2005) and this would then result in less overall pest resurgence. The question then is whether the stages that are invulnerable to some natural enemies are also invulnerable to other species of predators and parasitoids. For example, adult moths and butterflies are usually invulnerable to attacks by the natural enemies that attack their eggs and caterpillars, but they are attacked by other species, such as birds and bats, and there are indications that birds can be negatively affected by pesticides (Hallmann et al., 2014). Thus, side effects of pesticides should not only be studied for the natural enemies of the target pest stage, but also for the enemies of other pest stages. Furthermore, whether pesticide applications will result in more or less damage to the crop will also depend on the stage of the pest causing the damage: if this stage is vulnerable to natural enemies that are sensitive to pesticides, resurgence of this stage is predicted to occur, resulting in more damage.

### Conclusions

The majority of the studies reviewed concerned cases where native natural enemies were present locally (Figure 4), suggesting that this may be a general situation. However, exotic pests may invade areas where effective natural enemies are absent. In such cases, the use of pesticides may be the only remedy until suitable natural enemies are identified and released in biocontrol programs. The meta‐analysis did not include any cases of such release of natural enemies (van Lenteren, 2012) or cases where their efficiency was enhanced through targeted management practices (conservation biological control or functional agrobiodiversity, Gurr et al., 2017; van Rijn et al., 2013). Nevertheless, pest control by naturally occurring enemies was not less effective than chemical control in most cases (Figure 4b,d). This is in agreement with case studies that show that pesticide use can be decreased without loss of productivity (Wells et al., 2000; Seagraves & Lundgren, 2012; Lechenet et al., 2017; van Rijn et al., 2019, but see Zhang et al., 2015). Although more field experiments are needed to confirm the trends found here, both the theory and the meta‐analysis provide a novel argument to the proposition that research should aim at increasing the effectiveness of natural enemies, instead of focusing on the development and application of synthetic pesticides, with their reported disadvantages (Bryden et al., 2013; Gould et al., 2018; Halstead et al., 2014; Köhler & Triebskorn, 2013; Yamamuro et al., 2019). The effectiveness of natural enemies can be improved either by stimulating their occurrence through increasing habitat diversity (Holland et al., 2016; Rusch et al., 2016), by supplying alternative food resources to natural enemies (Albrecht et al., 2020; van Rijn et al., 2002, 2013), by introducing natural enemies of new, invasive pests or by augmentative biological control. This will be an important step in the redesign of productive, sustainable agricultural systems (Pretty, 2018; Wyckhuys et al., 2020).

## AUTHORSHIP

AJ conceived the study, AJ and PCJvR formulated the models, AJ ran model simulations, collected publications, did the meta‐analysis and prepared the figures. AJ and PCJvR prepared the manuscript.

### PEER REVIEW

The peer review history for this article is available at https://publons.com/publon/10.1111/ele.13819.

## Supporting information

Supplementary Material

## Data Availability

No original data were used in this manuscript, and all search terms and articles from the literature review are described and cited. Data used for the meta‐analysis are available at https://doi.org/10.21942/uva.14308868.v1.
