# Supplementary material for: Pesticides do not significantly reduce arthropod pest densities in the presence of natural enemies
Source: Ecol Lett. 2021 Jun 23;24(9):2010–24. doi: 10.1111/ele.13819 (PMC8453990; doi:10.1111/ele.13819)
Supplement: Supplementary file 1 — Supplementary Material [file ELE-24-2010-s001.pdf]

Supplementary Information, Figures and Tables for

Pesticides do not significantly reduce arthropod pest densities in the presence of natural enemies

Arne Janssen[1,2]* and Paul van Rijn[1]

[1]Institute for Biodiversity and Ecosystem Dynamics, Department of Evolutionary and Population Biology

University of Amsterdam, The Netherlands

[2]Department of Entomology, Federal University of Viçosa, Minas Gerais, Brazil

* Corresponding author. Email: arne.janssen@uva.nl

Table S1. Mortality rates of pests as result of pesticide applications in the field.

Study[a]	Pest	Active ingredient	survival	time (days)	Rate[b]
1.	*Sahlbergella singularis*	Thiametoxam	0.067	1	2.70
2.	*Bemisia tabaci*	Imidacloprid	0.6	2	0.26
3.	*Lygus hesperus*	Acetamiprid	0.143	5.5	0.35
3.	*Lygus hesperus*	Novaluron + Acetamiprid	0.368	5.5	0.18
3.	*Lygus hesperus*	Thiametoxam	0.330	5.5	0.20
3.	*Chaetosiphon fragaefolii* + *Myzus persicae*	Acetamiprid	0.214	5.5	0.28
3.	*Trialeurodes vaporariorum*	Acetamiprid	0.403	5.5	0.16
4.	*Acleris undulana*	Diflubenzuron	0.08	5	0.51
5.	*Chaetosiphon fragaefolii*	Acetamiprid	0.012	3	1.47
5.	*Chaetosiphon fragaefolii*	Abamectin	0.758	3	0.09
5.	*Chaetosiphon fragaefolii*	Pirimicarb	0.005	3	1.75
5.	*Lygus rugulipennis*	Thiacloprid	0.761	3	0.09
6.	*Frankliniella occidentalis*	Spinosad	0.282	2	0.63
6.	*Frankliniella occidentalis*	Bifenthrin	0.822	2	0.10
6.	*Frankliniella occidentalis*	Methomyl	0.962	2	0.02
6.	*Frankliniella occidentalis*	Methidathion	0.576	2	0.28
7.	*Leptinotarsa decemlineata*	Thiametoxam	0.341	3	0.36
8.	*Plutella xylostella*	Indoxacarb	0.54	1	0.62
9.	Jassids and lepidoptera	Deltamethrin	0.163	1	1.81
10.	*Bemisia tabaci*	Chloropyriphos + Cypermethrine	0.128	1	2.06
10.	*Empoasca devastance*	Chloropyriphos + Cypermethrine	0.123	1	2.10
10.	*Thrips tabaci*	Chloropyriphos + Cypermethrine	0.200	1	1.61

[a] Numbers refer to the following publications: 1. Anikwe *et al.* (2009); 2. Castle *et al.* (2014); 3. Dara (2016); 4. Erler *et al.* (2010); 5. Fitzgerald (2004); 6. Kay & Herron (2010); 7. Laznik *et al.* (2010); 8. Liu *et al.* (2003); 9. Macfadyen & Zaluki (2012); 10. Mamoon-ur-Rashid *et al.* (2012). [b] Daily per capita mortality rate, calculated as -ln(survival)/time.

Supplementary information S1. Further analysis of the basic predator-prey model

It is well-known that the original Rosenzweig-McArthur model can give rise to cycles of increasing amplitude with increasing carrying capacity *K* (the so-called paradox of enrichment Rosenzweig 1971). Adding constant pesticide-induced mortality can therefore stabilize the dynamics, but at higher pest levels. This can easily be seen from a phase-plane analysis of the model (Fig. S1).

In this figure, the prey isoclines are given in blue and the predator isoclines in orange. The vertical orange lines are the predator isoclines. It is well-known that the positive equilibrium is stable when this vertical orange predator isocline crosses to the right of the maximum of the blue parabola of the prey isocline (here marked by the green dot) and unstable if it crosses to the left (the orange dot). Because the isoclines change with pesticide application, the equilibrium can become stable with increasing pesticide-induced mortality. However, this also results in higher pest densities. The black dashed spiral to the right in the figure shows that the populations of the pest and natural enemy spiral from the initial densities (black dot) towards the stable equilibrium (green dot) after several fluctuations when pesticides are applied (p = 0.12). In contrast, without pesticides (p = 0), the dynamics spiral away from the unstable equilibrium (orange dot).

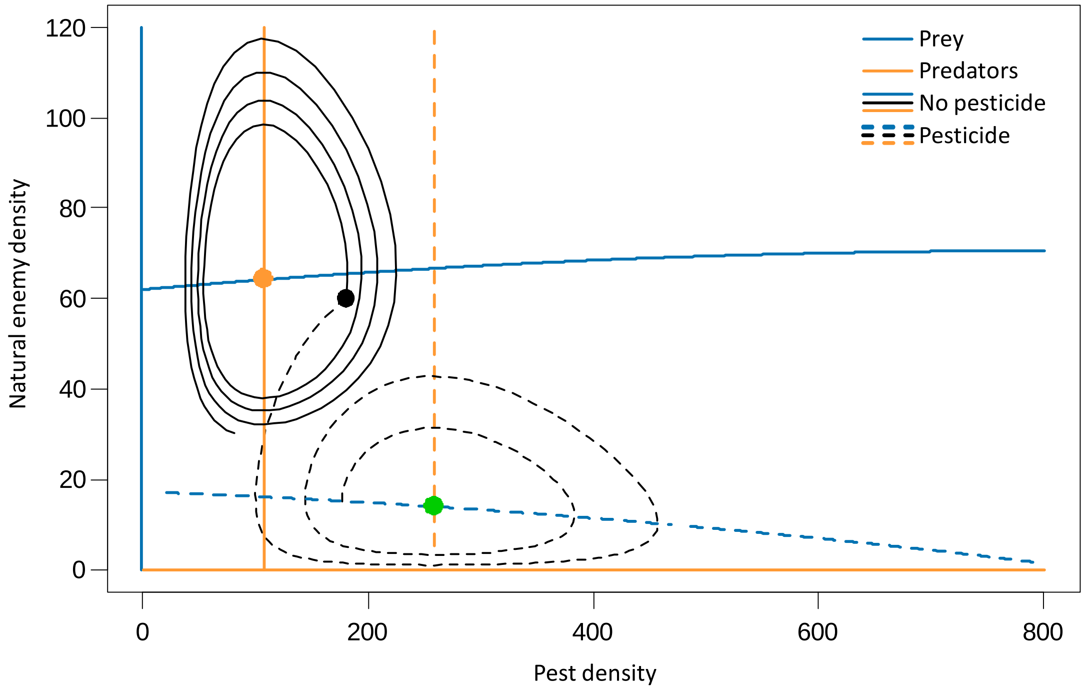

Fig. S1. Phase plane of the predator-prey model with stable and unstable dynamics (paradox of enrichment). Blue lines and curves are pest isoclines, orange are predator isoclines, solid lines are isoclines and trajectories without pesticide application, dashed lines with pesticide. The intercept of the blue pest isocline with the vertical orange predator isocline is the positive equilibrium where pest and natural enemies coexist, which is stable with pesticide application (green dot) and unstable without pesticides (orange dot). Black curves are trajectories of densities of pest and natural enemy through time, starting at the black dot. The solid black curve shows that densities spiral away from the unstable equilibrium (orange dot), the dashed black curve spirals towards the stable equilibrium (green dot). Parameter values are r = 0.166, c = 0.375, a = 4.0, m = 0.1, D = 1500, K = 3074.76; p = 0 or 0.13; q = 1.

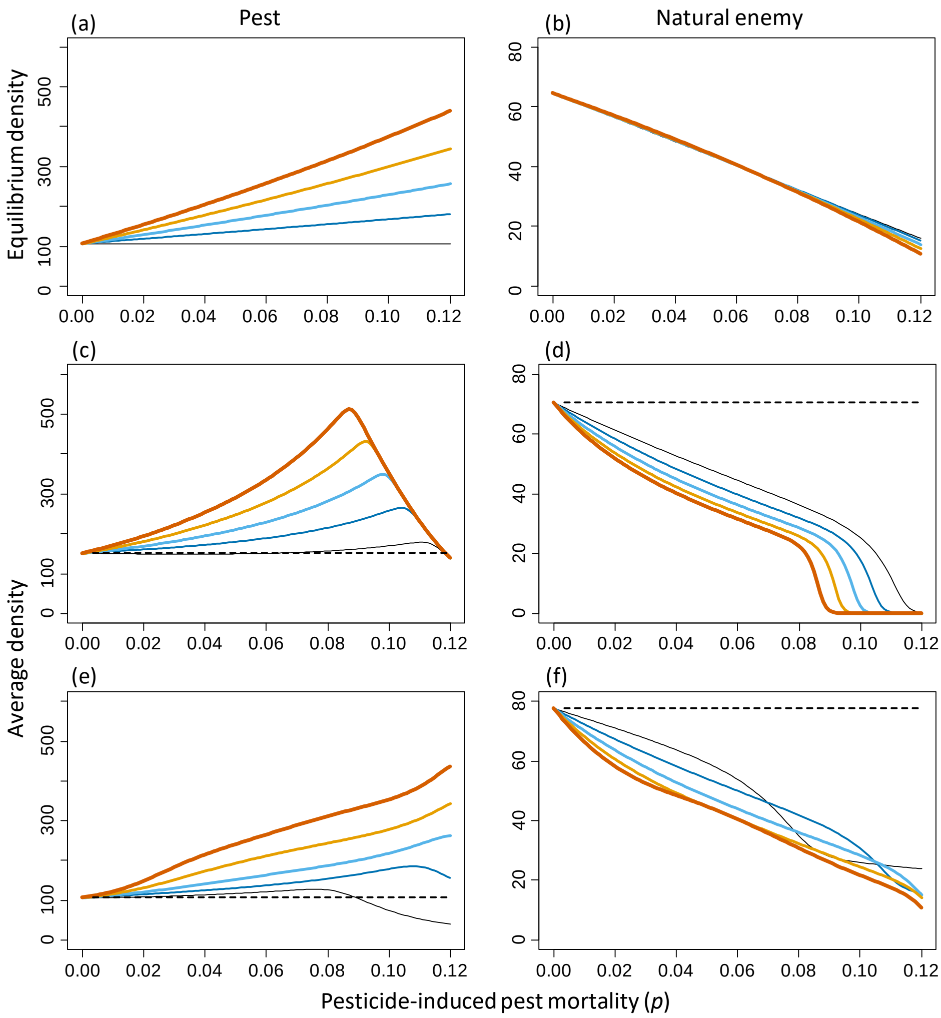

Fig. S2. Equilibrium pest (a) and natural enemy (b) densities of the simple predator-prey model with stable and unstable dynamics as function of the pesticide-induced pest (p) and natural enemy (q) mortality. Average pest (c, e) and natural enemy densities (d, f) during 5 pest generations. Pesticide-induced natural enemy mortality (q) varied from 0.0 to 2.0 relative to the pest mortality. (c-f) show average densities during 100 days when pesticide was applied continuously. Other parameter values are $r = 0.166$, $c = 0.375$, $a = 4.0$, $m = 0.1$, $D = 1500$, $K = 3074.76$, initial densities were 10 pest individuals per 1 natural enemy (c, d) or 400 pest individuals per 60 natural enemies (e, f).

The equilibrium densities of the pest do not change with increased pesticide-induced pest mortality if natural enemies do not suffer extra mortality because of the pesticides (thin black line in Fig S2a). When natural enemies do suffer from pesticide-induced mortality ($q > 0$), equilibrium pest densities go up with increasing pesticide-induced pest mortality (Fig. S2a, all lines except the black). Equilibrium densities of natural enemies go down with increasing pesticide-induced pest mortality (Fig S2b), even when the enemies are insensitive to the pesticide (Fig S2b, thin black curve).

We simulated the dynamics of the pest and natural enemies during five pest generations in the part of parameter space with a transition from an unstable ($p = 0$) to a stable equilibrium ($p = 0.12$). It shows that, although pesticide applications can stabilise the dynamics, this results in an increase of average pest densities (Fig. S2c, e) and a decrease of average densities of natural enemies (Fig. S2d, f). This figure also shows that equilibrium densities do not accurately reflect average densities of transient dynamics (compare Fig. S2a with c and e; b with d and f).

Fig. S3. Initial densities

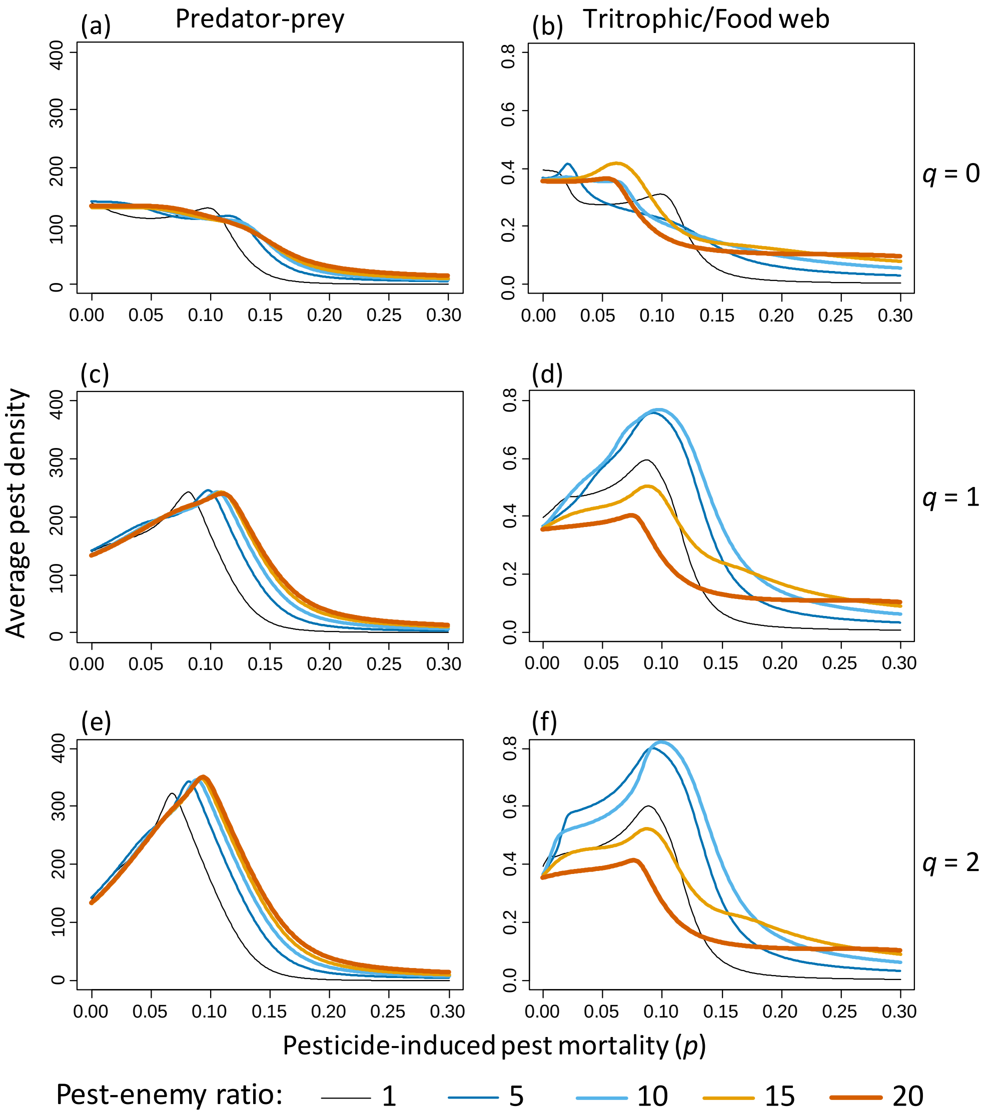

Fig. S3. Effects of pesticide-induced mortality (*p*, horizontal axis) on average pest densities (vertical axis) during five pest generations. Colours and thickness of curves indicate different initial pest-natural enemy ratios (ratios: black = 1 pest per enemy; dark blue = 5 (standard value); light blue =10; orange = 15; brown = 20). Increasing line thickness corresponds to increasing pest-enemy ratio. Left-hand column: basic predator-prey model; right-hand column: food web model without alternative prey. Pesticide-induced natural enemy mortality (*q*) was zero (a, b), equal to (c, d) or twice as high as that of the pest (e, f).

Supplementary information S2. Stage-structured model

Besides the basic predator-prey model and the tritrophic/food web model, we formulated a stage-structured model designed to capture the dynamics of an important plant pest (the Western flower thrips *Frankliniella occidentalis*) and one of its predators used to control it (the predatory mite *Iphiseius degenerans*), and was validated by greenhouse experiments (van Rijn *et al.* 2002). The version used here includes alternative food (pollen, *A*) for the predator only (not for the prey as in the original model, to make the model more general). The pest population was modelled as consisting of three stages: first-instar larvae that were vulnerable to predation (N_1), other juvenile instars (N_2) that were invulnerable to predation, and invulnerable reproducing adults (N_3). The predator population was also modelled in three stages: a non-predatory juvenile stage (P_1), a predatory juvenile stage (P_2) and a predatory adult stage (P_3), with the adult stage having the highest predation rate. We further modified the model by making reproduction of the pest a decreasing function of its density to avoid unlimited increases of the pest population in the absence of predators. The dynamics were modelled as follows:

$$\frac{dA}{dt} = a - bA - \frac{\Phi f_A}{N_1 + N_f + \Phi A + kAN_1} A(jP_2 + P_3)$$

$$\frac{dN_1}{dt} = rN_3 max\left[0, 1 - \frac{N_1 + N_2 + N_3}{C}\right] - \frac{f_N}{N_1 + N_f + \Phi A + kAN_1} N_1(jP_2 + P_3) - d_1N_1 - pN_1$$

$$\frac{dN_2}{dt} = d_1N_1 - d_2N_2 - pN_2$$

$$\frac{dN_3}{dt} = d_2N_2 - vN_3 - pN_3$$

$$\frac{dP_1}{dt} = \max\left[0, g\left(\frac{N_1 + \Phi A}{N_1 + \Phi A + N_g} - m\right)\right] P_3 - eP_1 - pqP_1$$

$$\frac{dP_2}{dt} = eP_1 - e_2P_2 - pqP_2$$

$$\frac{dP_3}{dt} = e_2P_2 - \min\left[\mu_0, \mu\left(\frac{N_1 + \Phi A + N_\mu}{N_1 + \Phi A}\right)\right] P_3 - pqP_3$$

Pollen (*A*) is produced at a constant rate (*a*), decays with rate *b*, and is consumed by predatory juveniles (P_2) and adults (P_3), with juveniles consuming at a fraction *j* of consumption rate of adult predators. Pollen consumption follows a type II saturating functional response with f_A being the maximum consumption rate by the predators, N_f the half-saturation density of vulnerable prey, Φ the food value of pollen relative to prey, and *k* the reduction in predation of the pest because of the presence of pollen (van Rijn *et al.* 2002). First-instar pest larvae (N_1) are produced by adults (N_3), with net per capita reproduction *r* being reduced at increasing densities of the pest, *C* is a constant determining the carrying capacity of the pest. The vulnerable stage is consumed by predators (P_2 and P_3) with a saturating functional response, in which f_N is the maximum predation rate and the other parameters (N_f, Φ , *k*) as above. All prey stages incur a pesticide-induced per capita mortality of *p* (varied). Surviving individuals develop into invulnerable juveniles with per capita rate d_1, which develop into adults with rate d_2. Adult pests have a natural mortality of *v* in addition to the pesticide-induced mortality *p*.

Predator juveniles, corrected for sex ratio and juvenile survival, are produced by adult predators (P_3) as a result of the consumption of pest individuals and of pollen, following a Michaelis-Menten function for substitutable food types (van Rijn *et al.* 2002):

$$\max\left[0, g\left(\frac{N_1+\Phi A}{N_1+\Phi A+N_g} - m\right)\right]$$

with *g* the maximum rate of reproduction, N_g the half-saturation pest density and *m* maintenance costs. Non-predatory juveniles develop into predatory juveniles with rate e_1. All predator stages experience pesticide-induced mortality *pq* as above. Predatory juveniles (P_2) develop into adults with rate e_2. The rate of development

of these juveniles into adults is independent of the food density, whereas the food-dependent juvenile survival is included in the net reproduction rate of the adults. Per capita adult predator (P_3) mortality increases with decreasing food availability, modelled as the inverse of a Michaelis-Menten function (van Rijn *et al.* 2002):

$$\min\left[\mu_0, \mu\left(\frac{N_1 + \Phi A + N_\mu}{N_1 + \Phi A}\right)\right]$$

with μ_0 the mortality at very low food densities, and μ that at very high densities and N_μ the pest density for which the mortality is half its maximum. Parameter values were a = 0.17; b = 0.186; Φ = 0.34; f_A = 0.085; N_f = 1.5; k = 0.04; j = 0.25; r=2.0; C = 112.4; f_N = 4.0; d_1 = 1/3; d_2 = 1/15; v = 0.11; g = 1.875; N_g = 1.0; m = 0.2; e = 1/3; e_2=1/5; μ_0 = 0.2; μ = 0.0625; N_μ = 0.08. See (van Rijn *et al.* 2002) for further details and units.

Pesticide applications were simulated as above, both in the absence and presence of the alternative food (pollen), with the following initial densities: A = 0 or 1; N_1 = 0.168; N_2 = 0.39; N_3 = 0.042; P_1 = 0; P_2 = 0; P_3 = 0.1). Threshold densities for application were 2 dm^{-2} (all pest stages combined). Simulations again lasted for five pest generations (c. 100 days).

Simulations with the stage-structured model showed the effects of differential vulnerability of pest stages to pest resurgence: only the pest stage vulnerable to predation showed some resurgence, whereas densities of the two invulnerable pest stages were reduced by the pesticide (Fig. S4a), resulting in a reduction of the overall pest density.

It should be noted that the vulnerable larval stage in this model is short (3 days) relative to the invulnerable larval (15 days) and adult stages (9 days). To further investigate the effect of the duration of the invulnerable stage, we exchanged the duration of the vulnerable larval stage (to 15 days) with that of the invulnerable larval stage (to 3 days), whereas the adult pests were still assumed invulnerable. This assumption of vulnerability of the stages of this pest holds for larger predators such as predatory bugs, which attack both larval stages (van den Meiracker & Sabelis 1999).

Increasing the duration of the vulnerable immature stage at the expense of that of the invulnerable immature stage resulted in increased pest resurgence, even with the adult stage being invulnerable to predation (Fig. S4b). Hence, pests with longer periods of vulnerability to natural enemies will more likely show pest resurgence with pesticide applications than pests with shorter vulnerable periods.

We again estimated the effects of pesticides on average pest densities over 5 generations by repeated simulations with varying pesticide-induced pest and natural enemy mortality and various pest application methods, as was done for the two models presented in the main text. The stage-structured model showed increases in average total pest densities (all stages combined) for intermediate values of pesticide-induced mortality for the pest and high mortality of the natural enemy (Fig. S5a, b). All other combinations of mortality of the pest and natural enemy resulted in lower average pest densities. Varying the pesticide application frequency with a pesticide-induced pest mortality (p) of 1 day^{-1}, resulted in no increase or decrease of average pest densities with low pesticide application frequencies (Fig. S5c), and an increase for higher frequencies and high natural enemy mortality (q > 1, Fig. S5c, d). Pesticides reduced average pest densities only with high application frequencies and low natural enemy mortality (q < 1), coinciding with the near extinction of natural enemies (Fig. S5d). Similar patterns in average pest and natural enemy densities as with pesticide applications every 2 weeks (Fig. S5a, b) were observed for threshold and continuous pesticide applications (Fig. S5e-h). In conclusion, compared to pests without stages that are invulnerable to natural enemies, pests with long invulnerable stages are better controlled with pesticides (compare Fig. 2 and 3 with S5).

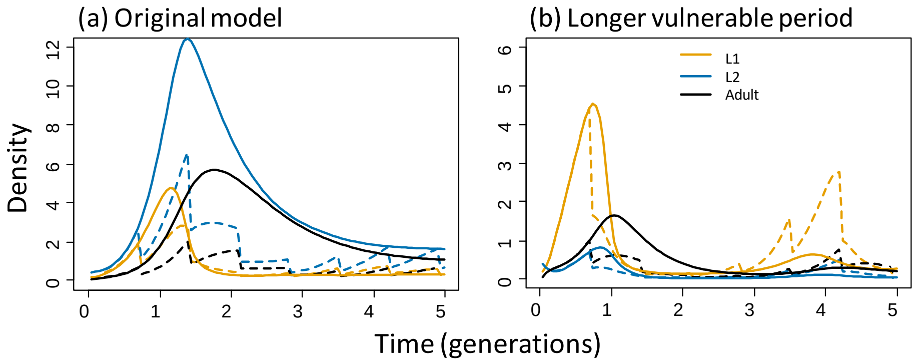

Fig. S4. Representative medium-term dynamics of pest and natural enemy densities (five pest generations). (a) A stage structured model consisting of a pest with a juvenile stage that is vulnerable to predation by the natural enemy (L1, orange), and invulnerable juvenile (L2, blue) and adult stages (black). All stages were sensitive to pesticides. Drawn curves are dynamics without pesticide, broken curves with pesticides. The vulnerable stage (orange curve) shows several short periods of pest resurgence (dashed orange curve above the drawn orange curve). (b) The same model as in (a)**.** but with a longer vulnerable juvenile period (15 d instead of 3 d) and a shorter invulnerable juvenile period (3 d instead of 15 d), showing pronounced pesticide resurgence after the third generation. Pesticide-induced pest mortality was p = 1 day^{-1} and natural enemy mortality half of that (q = 0.5). Pesticide was applied with a frequency of every 14 days.

With an increased period of pest vulnerability, pesticide application often did not result in decreased average pest densities (Fig. S6, most coloured curves at or above the dashed black line), and again resulted in increased pest densities when natural enemies suffered high mortality from the pesticide (Fig. S6, orange and brown curves). Comparison with Fig. S5 thus shows that the duration of the vulnerable period of the pest to natural enemies influences the effect of pesticides on pest densities; if natural enemies have a larger effect on pest densities (*i.e.* a longer vulnerable period), pesticides are less efficient in reducing pest densities.

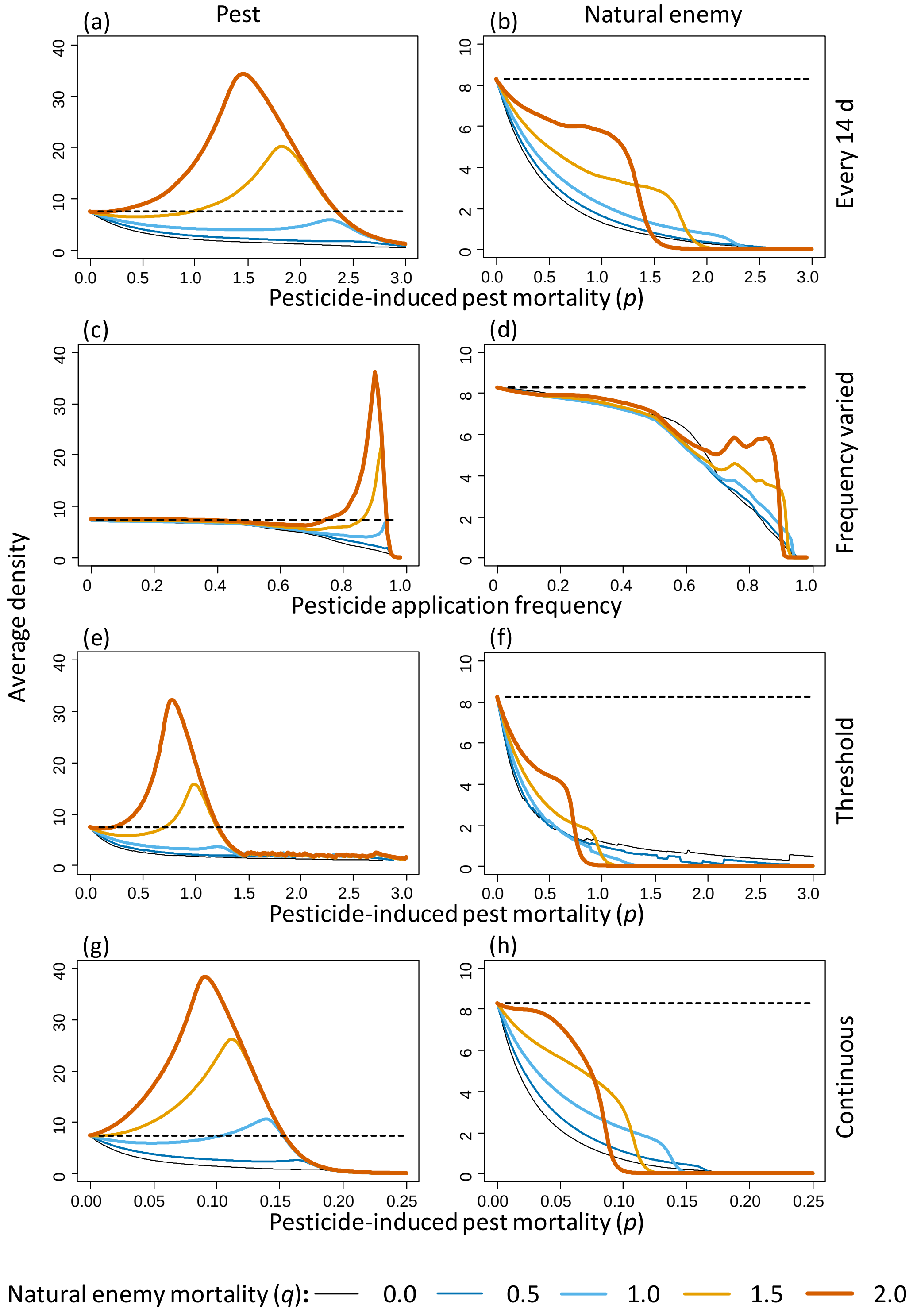

Fig. S5. Average pest (left column) and natural enemy (right column) densities (vertical axis) over 5 pest generations of the stage-structured predator-prey model with a short period of prey vulnerability (3 d) as a function of pesticide-induced pest mortality (p) or pesticide application interval (horizontal axis). Each row represents a different pesticide application method. See Fig. 2 for further explanation.

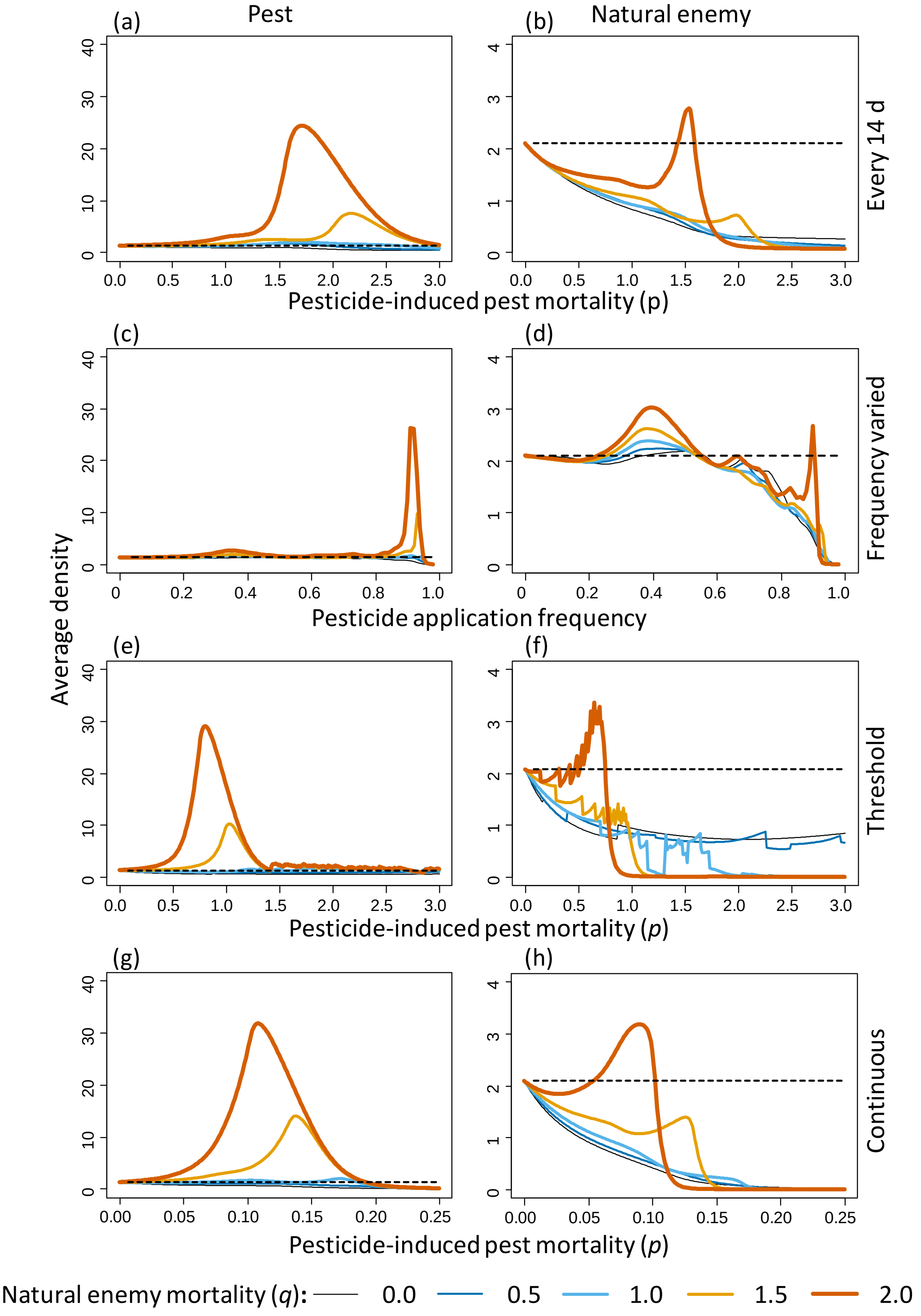

Fig. S6. Average pest (left column) and natural enemy (right column) densities of simulations of the stage-structured population model (5 generations) with a longer period of prey vulnerability (15 d) and a shorter invulnerable immature period (3 d). Densities are shown as a function of pesticide-induced pest mortality (*p*) or pesticide application interval (horizontal axis). Each row represents a different pesticide application method. See Fig. 2 for further explanation.

Table S2. Characteristics of studies included in the meta-analyses of time series of pests (A) and of average pest densities (B).

A. Studies with time series

**Study** [a]	**Crop**	**Pest**	**Enemy**	**Country** [b]	**Plot size**	**Pesticides (active ingredient)**
1	eggplant	*Tetranychus urticae*	*Phytoseiulus persimilis*	Syria	27 plants	Acetamiprid, Deltamethrin, Fenbutatin oxide
2	cassava	*Mononychellus progressivus*	*Amblyseius limonicus*	Colombia	5000 plants	Permethrin
3	soy bean	*Pseudoplusia includes* + *Anticarsia gemmatalis* [c]	Various hemipterans, coleopterans and parasitoids [d]	Brazil (Goias)	625 m^2	Cypermethrin, Imidacloprid, Beta-cyflutrin, Lambda-cyhalotrin, Thiametoxan, Endosulfan, Methamidophos, Methoxyfenozide, Permethrin
3	soy bean	*Pseudoplusia includes* + *Anticarsia gemmatalis* [c]	Various hemipterans, coleopterans and parasitoids [d]	Brazil (Paraná)	625 m^2	Methoxyfenozide, Acephate, Methamidophos, Permethrin, Imidacloprid, Beta-cyflutrin
4	cotton	*Amrasca biguttula biguttula*, *Bemisia tabaci*, *Helicoverpa armigera*	*Cheilomenes sexmaculatus*, *Chrysopa* spp., spiders	India (Andhra Pradesh)	4 x 4m rows	Imidacloprid, Methomyl, Endosulfan, Cypermethrin, Monocrotophos
5	lettuce	*Nasonovia ribisnigri* [e]	*Micromus tasmaniae*, *Coccinella undecimpunctata*, *Melanostoma fasciatum*	New-Zealand	24 m^2	Imidacloprid
6	okra	*Aphis gossypii*	*Menochilus sexmaculatus*, *Coccinella septempunctata*, *Oxyopes* sp., *Clubiona* sp.	India (Tamil Nadu)	16 m^2	Carbosulfan, Imidacloprid
7	cotton	*Aphis gossypii*	*Lysiphlebus testaceipes*, arthropod predators	USA (Alabama)	0.2 ha	Cypermethrin, Sulprofos
8	apple	*Aculus schlechtendali*, *Panonychus ulmi*, *Tetranychus urticae*	*Typhlodromus caudiglans*, *Zetzellia mali*	Canada (Ontario)	9 trees	Permethrin
9	cotton	*Adelphocoris suturalis*	Absent	China (Henan)	0.056 ha	b-Cypermethrin, Fipronil, Cyhalothrin, Endosulfan, Monocrotophos, Avermectins, Pyridaben, Imidacloprid

10	cotton	*Apolygus lucorum*	*Peristenus* spp, *Orius* spp., *Geocoris* spp., *Nabis* spp., Coccinellids[f]	China (Henan)	0.056 ha	b-Cypermethrin, Monocrotophos, Avermectins, Pyridaben, Cyhalothrin, Imidacloprid, Chlorantraniliprole
11	grape	*Tetranychus mcdanieli*	Various Phytoseiids	USA (Oregon)	plant	Chlorpyrifos, Chlorpyrifos + Sulfur
12	Soy bean	*Aphis glycines*	*Orius insidiusus, Nabis americoferus, Chrysoperla sp.,* Coccinellids	USA (S. Dakota)	40.3 m^2	Imidacloprid, Thiamethoxam
13	cotton	*Aphis gossypii*	Coccinellids [g]	USA (Georgia)	0.1 ha	Imidacloprid
14	alfalfa	*Acyrthosiphon pisum*, *Therioaphis trifolii*	Coccinellids, Lacewings, Syrphids, parasitoids	China (Gangsu)	667 m^2	Imidacloprid
15	cotton	*Aphis gossypii*	*Harmonia axyridis*, *Propylea japonica*	China (Shandong)	200 m^2	Clothianidin, Imidacloprid, Nitenpyram

B. Studies with long-term averages

Study	Crop	Pest	Enemy	Country	Plot size	Pesticides (active ingredients)
16	cotton	*Spodoptera exigua*	Various [h]	Pakistan	0.05 ha	Lufenuron, Chlorpyrifos
17	cotton	*Earias insulana*, *Pectinophora gossypiella*	Absent [i]	Pakistan	0.05 ha	Bifenthrin, Thiodicarb, Triazophos
18	apple	*Aculus schlechtendali*, *Aphis pomi*, *Eriosoma lanigerum*, *Panonychus ulmi* + *Tetranychus urticae* + *Tetranychus mcdanieli*	*Aphelinus mali*, *Forficula auricularia*, Phytoseiids	USA (Wash)	0.35 ha	Chlorantraniliprole, Methoxyfenozide, Novaluron, Spinetoram
19	cotton	*Aphis gossypii*	Coccinellids, Neuroptera, Spiders	China (Hebei)	400 m^2	b-Cypermethrin, Phoxim
20	cotton	*Helicoverpa armigera*, *Amrasca biguttula biguttula*, *Adelphocoris suturalis*	Various for *H. armigera*[i], Unknown for *A. biguttula,* None for *A. suturalis* [f]	China (Henan)	0.4 ha	Cypermethrin, Phosalone, Cypermethrin, Omethoate, Phosalone, Cypermethrin, Omethoate, Carbaryl

21	eggplant	*Amrasca biguttula biguttula*, *Aphis gossypii*, *Bemisia tabaci*, *Tetranychus urticae*, *Thrips palmi*	*Coccinella* sp., *Oxyopes* sp., *Tetragnatha* sp., *Homaeotarsus* sp., *Zelus* sp. *Ophionia nigrofasciata*, *Syrphus* sp.	Bangladesh	72 m^2	Imidacloprid, Emamectin benzoate
22	tomato	*Tetranychus marianae*	Unknown	USA (Guam)	64 m^2	Azadirachtin, Carbaryl, Malathion
23	potato	*Myzus persicae*, *Empoasca* sp., *Frankliniella occidentalis* + *Thrips tabaci*	Spiders, *Geocorus* sp., *Nabid* sp, *Orius* sp.	USA (Oregon)	337 m^2	Permethrin, Phorate, Disulfoton
24	walnut	*Chromaphis juglandicola*, *Panaphis juglandis*, *Panonychus ulmi*, *Tetranychus urticae*	*Trioxys pallidus*, Phytoseiids, *Chrysoperla* spp., Syrphinae	USA (California)	0.4 ha, 0.61 ha	Chlorantraniliprole, Lambda-cyalothrin, Chloroantraniliprole, Spinetoram, Chlopyrifos
25	citrus	*Phyllocnistis citrella*	Various [j]	USA (Florida)	4 trees	Cyantraniliprole, Fenpropathrin, Thiametoxam

[a] Numbers refer to the following publications: 1: Barbar (2017); 2: Braun *et al.* (1987); 3: Bueno *et al.* (2011); 4: Dhillon *et al.* (2012); 5: Fagan *et al.* (2010); 6: Gandhi *et al.* (2006); 7: Kerns & Gaylor (1993); 8: Lester *et al.* (1998); 9: Li *et al.* (2010); 10: Li *et al.* (2011); 11: Prischmann *et al.* (2005); 12: Seagraves & Lundgren (2012); 13: Wells *et al.* (2000); 14: Yang *et al.* (2015); 15: Zhang *et al.* (2015); 16: Arshad & Suhail (2011); 17: Arshad *et al.* (2015); 18: Beers *et al.* (2016); 19: Lu *et al.* (2012); 20: Men *et al.* (2005); 21: Prodhan *et al.* (2018); 22: Reddy & Miller (2014); 23: Reed *et al.* (2001); 24: Shearer *et al.* (2016); 25: Tiwari & Stelinski (2013).

[b] States or provinces are given between brackets; [c] Species separated by a '+' were counted and reported together in the original publication and were analysed as such; [d] Hoffmann-Campo *et al.* (2000); [e] Authors report aphid densities, consisting mainly, but not exclusively of the species mentioned here; [f] Luo *et al.* (2014); [g] Wells *et al.* (2001); [h] https://www.cabi.org/isc/datasheet/53078#toDistributionMaps and http://uaf.edu.pk/uaf_research/prj_32.html; [i] www.cabi.org;
[j] http://entnemdept.ufl.edu/creatures/citrus/citrus_leafminer.htm.

Supplementary information S3. Extended analysis of the literature

Insufficient data were available to compare the effects among individual pesticides or substance groups. We used the Pesticides Properties Database of the University of Hertfordshire (Lewis *et al.* 2016) to assess the activity of the pesticides against the pests investigated. In case of doubt, we sought additional publications using "chemical control" and the scientific name of the pest species as search terms. We identified 9 out of 68 time series in which the activity of the pesticide against the pest was not confirmed. Removing these time series from the data set did not change the results of the meta-analysis in a major way: (natural enemies present locally: effect size = -0.73, conf. int. -1.50 to 0.044; regionally: -0.49, -0.75 to -0.24; no enemies: -1.31, -1.97 to -0.65, cf. Fig. 3b overall effects). Of the studies presenting average pest densities, activity of the pesticide against the pest in question was not confirmed in slightly less than half the cases (47). Upon removing these cases from the data set, meta-analysis results changed somewhat: the overall effect of pesticide use on pest densities still did not differ from zero in the presence of natural enemies locally (effect size: 0.58, conf. int. -0.20 to 1.35) and still did differ from zero when natural enemies were absent (-10.1, -17.5 – -2.76), but was no longer different from zero when enemies were present regionally (-1.18, -2.89 to 0.522) (cf. Fig. 3d overall effects). In conclusion, excluding cases where the effect of the pesticide on the pest could not be confirmed did not change our main conclusions in any way.

We assessed the presence of influential cases in both data sets, using Cook's distances and hat values to identify outliers (Viechtbauer 2010). In both data sets, one study contained several influential cases. For the time series data, removal of this study from the analysis resulted in the same patterns of significant and non-significant effect sizes as did the analysis of the full data set. After removing the outlier study from the data set of averages, the same results were found as with the full data set, except that the overall effect with natural enemies present regionally did no longer differ significantly from zero (cf. Fig. 4d).

One concern with meta-analysis is the publication bias. The idea is that studies showing non-significant results are less likely to be published (Rosenthal 1979). To estimate the potential effect of this, a simple technique is to calculate the number of studies with a zero effect size that need to be added to render the overall effect just not significantly different from zero (Rosenthal 1979). We calculated these so-called fail-safe numbers for the significant effects shown in Fig. 3 in the main text, showing that most significant effects seem relatively robust for such a publication bias (Table S3). To the best of our knowledge, a test for the opposite, i.e. how many cases should be added to render an effect size significantly different from zero, does not exist because no publication bias is expected for studies showing significant effects. This is perhaps not entirely true for the studies reviewed here: many researchers would probably expect an overall negative effect of pesticide applications on pest densities and would not be keen to publish positive effects. Hence, the lack of a significant overall negative effect of pesticides on pest densities strongly suggests that this is indeed a true phenomenon. However, if anything, our literature search shows that there is a general lack of studies on the effects of pesticide applications through time.

We also analysed the effect of the experimental plot sizes of those studies for which plot size was given on a square-meter scale. Because there were not sufficient studies for a full factorial analysis, we analysed a model with the presence of natural enemies, pesticide application method, time in generations and plot size (in m^2, Table S2) as factors without their interactions. The random factors and further model structure was similar to previous analyses. The effect of plot size was not significant for the time series of repeated measure or for the studies with long-term averages (LRT = 0.16, d.f. = 1, p = 0.69 and LRT = 0.59, d.f. = 1, p = 0.44, respectively).

Table S3. Fail-safe numbers of significant effects in Fig. 4b, d.

Enemy presence	Pesticide application	N studies (data points)	Significance [a] (**negative**/ positive)	Fail safe numbers
		**Time series**		
Local	Threshold	4 (26)	0.027	100
	Seed	12 (57)	**0.001**	3523
Regional	Overall	14 (147)	**0.002**	2449
	Regular	9 (101)	**0.006**	1461
	Threshold	5 (46)	**0.009**	85
None	Overall	5 (66)	**0.005**	2956
	Regular	4 (58)	**0.011**	1839
	Once	1 (8)	**0.005**	123
		**Averages**		
Regional	Overall	14 (14)	**0.040**	155
None	Overall	14 (14)	**0.008**	222
	Regular	6 (6)	**0.003**	56

[a] Significant negative effects are given in bold, significant positive effects in normal type.

Fig. S7. Natural enemy densities of the food-web model with alternative food.

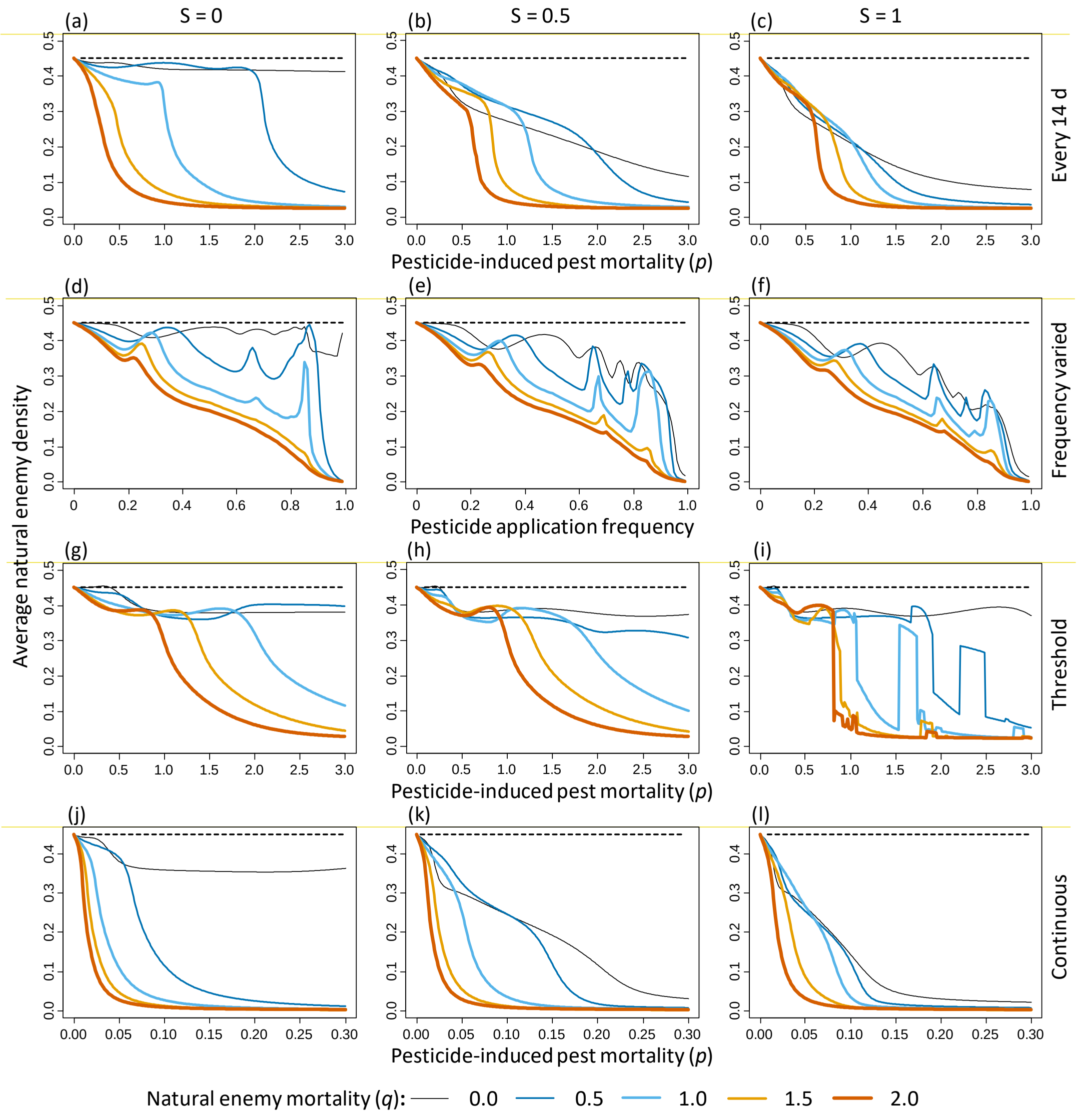

Fig. S7. Average natural enemy densities of simulations of the tritrophic/food web population model (5 generations) with an alternative prey. Average pest densities are shown in Fig. 5. Shown are average natural enemy densities with different levels of pesticide-induced mortality of the alternative prey: no mortality (*s* = 0, left-hand column), half that of the pest (*s* = 0.5, middle column) or the same as that of the pest (*s* = 1, right-hand column). See legend to Fig. 5 for further explanation.

Supplementary information S4. Alternative food in the stage-structured model

We assume here that alternative food does not change in quality because of pesticide applications. It partly compensates for the reduction of pest densities for the natural enemies due to pesticide applications, and this results in lower pest densities than without alternative food (cf. Fig. S8 and S4). The pest, however, still reaches higher densities than without pesticides for high mortality rates of the natural enemies ($q > 1$) (Fig. S8).

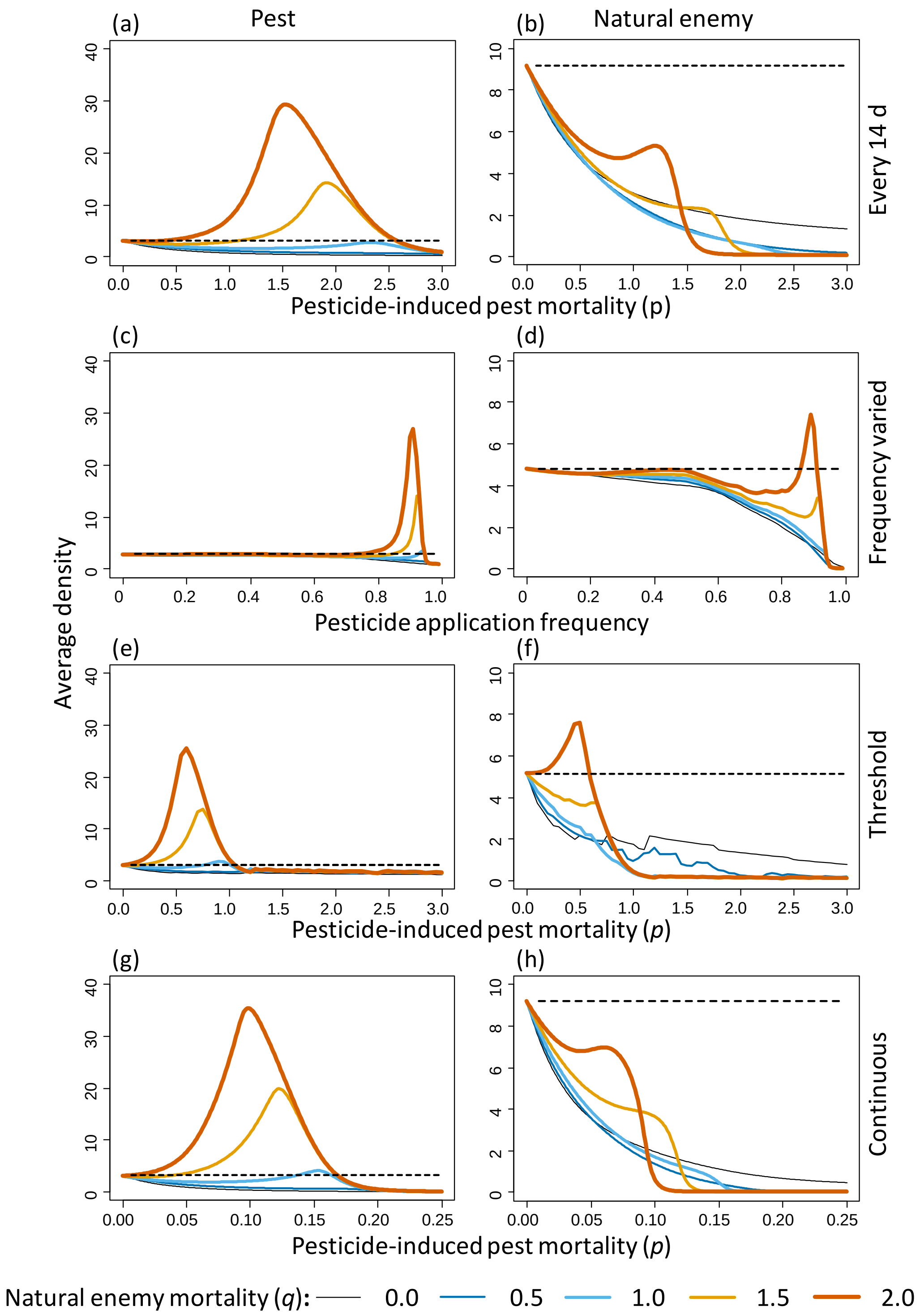

Fig. S8. Average densities of the pest (left column) and natural enemies (right column) of simulations of the stage-structured population model (5 generations) with pollen as alternative food for the natural enemies. Per panel, curves with different colours and thickness refer to different pesticide-induced natural enemy mortality relative to pest mortality: thin, black: q = 0; blue: q = 0.5; light blue: q = 1; orange: q = 1.5; thick, brown: q = 2. Increasing line thickness corresponds to increasing enemy mortality. The pest and enemy densities obtained without pesticide application are given as reference by black dashed horizontal lines. See legend to Fig. 2 for further explanation.

References

Anikwe, J., Asogwa, E., Ndubuaku, T. & Okelana, F. (2009). Evaluation of the toxicity of Actara 25 WG for the control of the cocoa mirid *Sahlbergella singularis* Hagl.(Hemiptera: Miridae) in Nigeria. *Afr. J. Biotechnol.*, 8.

Arshad, M., Gogi, M.D., Arif, M.J., Khan, R.R. & others. (2015). Seasonal pattern of infestation by spotted bollworm *Earias insulana* (Boisd.) and pink bollworm *Pectinophora gossypiella* (Saund.) in field plots of transgenic Bt and non-Bt cottons. *Pak. J. Zool.*, 47, 177–186.

Arshad, M. & Suhail, A. (2011). Field and laboratory performance of transgenic Bt cotton containing Cry1Ac against beet armyworm larvae (Lepidoptera: Noctuidae). *Pak. J. Zool.*, 43, 529–535.

Barbar, Z. (2017). Evaluation of three pesticides against phytophagous mites and their impact on phytoseiid predators in an eggplant open-field. *Acarologia*, 57, 529–539.

Beers, E.H., Horton, D.R. & Miliczky, E. (2016). Pesticides used against *Cydia pomonella*lu disrupt biological control of secondary pests of apple. *Biol. Control*, 102, 35–43.

Braun, A., Guerrero, J., Bellotti, A. & Wilson, L. (1987). Relative toxicity of permethrin to *Mononychellus progresivus* Doreste and *Tetranychus urticae* Koch (Acari: Tetranychidae) and their predators *Amblyseius limonicus* Garman & McGregor (Acari: Phytoseiidae) and *Oligota minuta* Cameron (Coleoptera: Staphylinidae): bioassays and field validation. *Environ. Entomol.*, 16, 545–550.

Bueno, A. de F., Batistela, M.J., Bueno, R. de F., França, N., Nishikawa, M.A.N., Liberio Filho, A., *et al.* (2011). Effects of integrated pest management, biological control and prophylactic use of insecticides on the management and sustainability of soybean. *Crop Prot.*, 30, 937–945.

Castle, S.J., Merten, P. & Prabhaker, N. (2014). Comparative susceptibility of *Bemisia tabaci* to imidacloprid in field-and laboratory-based bioassays. *Pest Manag. Sci.*, 70, 1538–1546.

Dara, S.K. (2016). Managing strawberry pests with chemical pesticides and non-chemical alternatives. *Int. J. Fruit Sci.*, 16, 129–141.

Dhillon, M.K., Pampapathy, G., Wadaskar, R. & Sharma, H. (2012). Impact of Bt transgenic cottons and insecticides on target and non-target insect pests, natural enemies and seedcotton yield in India. *Indian J. Agric. Sci.*, 82, 248–254.

Erler, F., Cetin, H., Saribasak, H. & Serttas, A. (2010). Laboratory and field evaluations of some botanical pesticides against the cedar leaf moth, *Acleris undulana*. *J. Pest Sci.*, 83, 265–272.

Fagan, L., McLachlan, A., Till, C. & Walker, M. (2010). Synergy between chemical and biological control in the IPM of currant-lettuce aphid (*Nasonovia ribisnigri*) in Canterbury, New Zealand. *Bull. Entomol. Res.*, 100, 217–223.

Fitzgerald, J. (2004). Laboratory bioassays and field evaluation of insecticides for the control of *Anthonomus rubi*, *Lygus rugulipennis* and *Chaetosiphon fragaefolii*, and effects on beneficial species, in UK strawberry production. *Crop Prot.*, 23, 801–809.

Gandhi, P.I., Gunasekaran, K. & Sa, T. (2006). Neem oil as a potential seed dresser for managing Homopterous sucking pests of Okra (*Abelmoschus esculentus* (L.) Moench). *J. Pest Sci.*, 79, 103–111.

Hoffmann-Campo, C.B., Moscardi, F., Corrêa-Ferreira, B.S., Oliveira, L.J., Sosa-Gómez, D.R., Panizzi, A.R., *et al.* (2000). *Pragas da soja no Brasil e seu manejo integrado* (No. 30). Embrapa Soja Londrina.

Kay, I.R. & Herron, G.A. (2010). Evaluation of existing and new insecticides including spirotetramat and pyridalyl to control *Frankliniella occidentalis* (Pergande)(Thysanoptera: Thripidae) on peppers in Queensland. *Aust. J. Entomol.*, 49, 175–181.

Kerns, D. & Gaylor, M. (1993). Induction of cotton aphid outbreaks by insecticides in cotton. *Crop Prot.*, 12, 387–393.

Laznik, Ž., Tóth, T., Lakatos, T., Vidrih, M. & Trdan, S. (2010). Control of the Colorado potato beetle (*Leptinotarsa decemlineata* [Say]) on potato under field conditions: a comparison of the efficacy of foliar application of two strains of *Steinernema feltiae* (Filipjev) and spraying with thiametoxam. *J. Plant Dis. Prot.*, 117, 129–135.

Lewis, K.A., Tzilivakis, J., Warner, D.J. & Green, A. (2016). An international database for pesticide risk assessments and management. *Hum. Ecol. Risk Assess. Int. J.*, 22, 1050–1064.
Li, G., Feng, H., Chen, P., Wu, S., Liu, B. & Qiu, F. (2010). Effects of transgenic Bt cotton on the population density, oviposition behavior, development, and reproduction of a nontarget pest, *Adelphocoris suturalis* (Hemiptera: Miridae). *Environ. Entomol.*, 39, 1378–1387.
Li, G., Feng, H., McNeil, J.N., Liu, B., Chen, P. & Qiu, F. (2011). Impacts of transgenic Bt cotton on a non-target pest, *Apolygus lucorum* (Meyer-Dür) (Hemiptera: Miridae), in northern China. *Crop Prot.*, 30, 1573–1578.
Liu, T.-X., Sparks, A.N. & Chen, W. (2003). Toxicity, persistence and efficacy of indoxacarb and two other insecticides on *Plutella xylostella* (Lepidoptera: Plutellidae) immatures in cabbage. *Int. J. Pest Manag.*, 49, 235–241.
Lu, Y., Wu, K., Jiang, Y., Guo, Y. & Desneux, N. (2012). Widespread adoption of Bt cotton and insecticide decrease promotes biocontrol services. *Nature*, 487, 362–365.
Luo, S., Naranjo, S.E. & Wu, K. (2014). Biological control of cotton pests in China. *Biol. Control*, 68, 6–14.
Macfadyen, S. & Zalucki, M.P. (2012). Assessing the short-term impact of an insecticide (Deltamethrin) on predator and herbivore abundance in soybean *Glycine max* using a replicated small-plot field experiment. *Insect Sci.*, 19, 112–120.
Mamoon-ur-Rashid, M., Khattak, M.K. & Abdullah, K. (2012). Evaluation of botanical and synthetic insecticides for the management of cotton pest insects. *Pak. J. Zool.*, 44, 1317–1324.
van den Meiracker, R.A.F. & Sabelis, M.W. (1999). Do functional responses of predatory arthropods reach a plateau? A case study of *Orius insidiosus* with Western flower thrips. *Entomol. Exp. Appl.*, 90, 323–329.
Men, X., Ge, F., Edwards, C.A. & Yardim, E.N. (2005). The influence of pesticide applications on *Helicoverpa armigera* Hübner and sucking pests in transgenic Bt cotton and non-transgenic cotton in China. *Crop Prot.*, 24, 319–324.
Prischmann, D.A., James, D.G., Wright, L.C., Teneyck, R.D. & Snyder, W.E. (2005). Effects of chlorpyrifos and sulfur on spider mites (Acari: Tetranychidae) and their natural enemies. *Biol. Control*, 33, 324–334.
Prodhan, M., Hasan, M., Chowdhury, M., Alam, M., Rahman, M., Azad, A., *et al.* (2018). Bt eggplant (*Solanum melongena* L.) in Bangladesh: Fruit production and control of eggplant fruit and shoot borer (*Leucinodes orbonalis* Guenee), effects on non-target arthropods and economic returns. *PloS One*, 13, e0205713redd.
Reddy, G.V. & Miller, R.H. (2014). Biorational versus conventional insecticides – Comparative field study for managing red spider mite and fruit borer on tomato. *Crop Prot.*, 64, 88–92.
Reed, G.L., Jensen, A.S., Riebe, J., Head, G. & Duan, J.J. (2001). Transgenic Bt potato and conventional insecticides for Colorado potato beetle management: comparative efficacy and non-target impacts. *Entomol. Exp. Appl.*, 100, 89–100.
van Rijn, P.C.J., van Houten, Y.M. & Sabelis, M.W. (2002). How plants benefit from providing food to predators even when it is also edible to herbivores. *Ecology*, 83, 2664–2679.
Rosenthal, R. (1979). The file drawer problem and tolerance for null results. *Psychol. Bull.*, 86, 638.
Rosenzweig, M.L. (1971). Paradox of enrichment: destabilization of exploitation ecosystems in ecological time. *Science*, 171, 385–387.
Seagraves, M.P. & Lundgren, J.G. (2012). Effects of neonicitinoid seed treatments on soybean aphid and its natural enemies. *J. Pest Sci.*, 85, 125–132.
Shearer, P.W., Amarasekare, K.G., Castagnoli, S.P., Beers, E.H., Jones, V.P. & Mills, N.J. (2016). Large-plot field studies to assess impacts of newer insecticides on non-target arthropods in Western U.S. orchards. *Biol. Control*, 102, 26–34.
Tiwari, S. & Stelinski, L.L. (2013). Effects of cyantraniliprole, a novel anthranilic diamide insecticide, against Asian citrus psyllid under laboratory and field conditions. *Pest Manag. Sci.*, 69, 1066–1072.

Viechtbauer, W. (2010). Conducting meta-analyses in R with the metafor package. *J. Stat. Softw.*, 36, 1–48.
Wells, M.L., McPherson, R.M., Ruberson, J.R. & Herzog, G.A. (2000). Effect of fungicide application on activity of *Neozygites fresenii* (Entomophthorales: Neozygitacaea) and cotton aphid (Homoptera: Aphididae) suppression. *J. Econ. Entomol.*, 93, 1118–1126.
Wells, M.L., McPherson, R.M., Ruberson, J.R. & Herzog, G.A. (2001). Coccinellids in cotton: Population response to pesticide application and feeding response to cotton aphids (Homoptera : Aphididae). *Environ. Entomol.*, 30, 785–793.
Yang, Q., Liu, C. & Zhang, Y. (2015). Mowing versus insecticide for control of alfalfa aphids and their differential impacts on natural enemies. *Egypt. J. Biol. Pest Control*, 25, 285.
Zhang, P., Zhang, X., Zhao, Y., Ren, Y., Mu, W. & Liu, F. (2015). Efficacy of granular applications of clothianidin and nitenpyram against *Aphis gossypii* (Glover) and *Apolygus lucorum* (Meyer-Dür) in cotton fields in China. *Crop Prot.*, 78, 27–34.
